# Deciphering the Underlying Mechanisms of Formula Le-Cao-Shi Against Liver Injuries by Integrating Network Pharmacology, Metabonomics, and Experimental Validation

**DOI:** 10.3389/fphar.2022.884480

**Published:** 2022-04-25

**Authors:** Qing Zhao, Xia Ren, Shu-Yue Song, Ri-Lei Yu, Xin Li, Peng Zhang, Chang-Lun Shao, Chang-Yun Wang

**Affiliations:** ^1^ Key Laboratory of Marine Drugs, The Ministry of Education of China, School of Medicine and Pharmacy, Ocean University of China, Qingdao, China; ^2^ Laboratory for Marine Drugs and Bioproducts, Qingdao National Laboratory for Marine Science and Technology, Qingdao, China

**Keywords:** traditional Chinese medicine, formula Le-Cao-Shi, liver injury, network pharmacology, metabonomics

## Abstract

Le-Cao-Shi (LCS) has long been used as a folk traditional Chinese medicine formula against liver injuries, whereas its pharmacological mechanisms remain elusive. Our study aims to investigate the underlying mechanism of LCS in treating liver injuries *via* integrated network pharmacology, metabonomics, and experimental validation. By network pharmacology, 57 compounds were screened as candidate compounds based on ADME parameters from the LCS compound bank (213 compounds collected from the literature of three single herbs). According to online compound–target databases, the aforementioned candidate compounds were predicted to target 87 potential targets related to liver injuries. More than 15 pathways connected with these potential targets were considered vital pathways in collectively modulating liver injuries, which were found to be relevant to cancer, xenobiotic metabolism by cytochrome P450 enzymes, bile secretion, inflammation, and antioxidation. Metabonomics analysis by using the supernatant of the rat liver homogenate with UPLC-Q-TOF/MS demonstrated that 18 potential biomarkers could be regulated by LCS, which was closely related to linoleic acid metabolism, glutathione metabolism, cysteine and methionine metabolism, and glycerophospholipid metabolism pathways. Linoleic acid metabolism and glutathione metabolism pathways were two key common pathways in both network pharmacology and metabonomics analysis. In ELISA experiments with the CCl_4_-induced rat liver injury model, LCS was found to significantly reduce the levels of inflammatory parameters, decrease liver malondialdehyde (MDA) levels, and enhance the activities of hepatic antioxidant enzymes, which validated that LCS could inhibit liver injuries through anti-inflammatory property and by suppressing lipid peroxidation and improving the antioxidant defense system. Our work could provide new insights into the underlying pharmacological mechanisms of LCS against liver injuries, which is beneficial for its further investigation and modernization.

## 1 Introduction

The liver is a vital organ that plays a crucial role in the normal metabolic homeostasis of the body, including the biotransformation of food, drugs, and endogenous and exogenous substances (Lahon and Das, 2011). Primary liver injuries caused by dangerous xenobiotics and metabolic burden could finally lead to serious liver diseases (Zhao et al., 2019; Hui et al., 2020). It was reported that more than 10% of the populations worldwide are suffering from liver injuries (Li et al., 2018). Therefore, developing effective drugs to prevent and treat liver injuries is urgently needed.

Traditional Chinese herbal formulae have been gaining more and more attention worldwide due to their moderate treatment methods and lower side effects when treating diseases (Zimmermann et al., 2007; Venkatesha et al., 2011). One formula tends to contain numerous bioactive ingredients, and therefore, it treats diseases in a holistic manner with multiple targets and pathways involved, which makes it challenging to decode the intricate mechanism, especially *via* conventional TCM study methodologies (Zhang Y. et al., 2014; Tang et al., 2015; Yuan et al., 2017). Fortunately, the advent of the era of big data, such as the high-throughput data and tremendously rich information provided by “Omics” technologies, promoted the “dry” experiment (*in silico* approach) featured with a high-throughput, cost-effective, and time-saving strategy, which is becoming more and more popular (Huang et al., 2013). Specifically, system biology, featured with non-reductive and holistic characteristics, could help decipher the complex mechanisms of formula (Zhang J. et al., 2014). Based on system biology, network pharmacology, as a paradigm in drug study, has been promptly used to interpret the mechanism of TCM at the molecular network level (Gu et al., 2011). Network pharmacology could comprehensively and systematically analyze the interactions between chemical constituents, target proteins, and pathways of TCM using existing databases (Chen et al., 2016; Yuan et al., 2017). Moreover, the application of network pharmacology to TCM may help discover potential molecular drugs (Chen et al., 2016). Metabonomics, a research frontier of systems biology, is a valuable strategy to capture the metabolic alterations of endogenous metabolites with low molecular weight in a biological system through modern analytical techniques and explore the intervention mechanism of TCM (Naz et al., 2014; Pirhaji et al., 2016; Geng et al., 2017; Sun et al., 2018). The metabonomics method could provide a global overview of multiple biochemical pathways to uncover disease pathogenesis and mechanisms of multicomponent medicinal effects. Nowadays, increasing attention has been focused on metabonomics to successfully reveal the mechanisms of TCM prescriptions (Wang et al., 2010; Patti et al., 2012; Cui et al., 2017; Gao et al., 2018; Pang et al., 2018; Wu et al., 2018; Wang et al., 2020; Li et al., 2021).

Formula Le-Cao-Shi (LCS) is an effective TCM in the treatment of liver injuries caused by chemical toxins or viruses (Ren et al., 2019; Zhao et al., 2019). It consists of three kinds of medicinal plants, namely, *Acanthus ilicifolius* L., *Phyllodium pulchellum* (L.) Desv., and *Cudrania cochinchinensis* Lour. (or *C. tricuspidata* (Carr.) Bur.) in a ratio of 5:2:3 (Guan and Wang, 2009; Guan and Wang, 2013). Among them, *A. ilicifolius* Linn. is a mangrove plant growing in tropical and subtropical intertidal habitats. Pharmacological research has demonstrated that its extracts possess multiple bioactivities, including hepatoprotective, antiviral, antioxidant, and anti-inflammatory activities (Guan and Wang, 2009; Guan and Wang, 2013; Guan and Wang, 2015; Wei et al., 2015). *P. pulchellum* (L.) Desv. is a shrub belonging to the family Papilionoideae, distributed in Southern China and India (State Administration of Traditional Chinese Medicine, 1999a; Editorial Board of Flora of China, 2010). It has been reported that the extracts of its aerial part exhibited significant antifibrotic activity and could be used to treat the enlargement of the liver and spleen, rheumatism, bone pain, and swelling (Fan et al., 2018). *C. cochinchinensis* Lour. is also a shrub belonging to the family Moraceae (State Administration of Traditional Chinese Medicine, 1999b). Its radix has been used as a remedy to treat hepatitis and rheumatism (State Administration of Traditional Chinese Medicine, 1999b; Xin et al., 2017). *C. tricuspidata*, the alternative herb of *C. cochinchinensis*, is difficult to obtain due to its relatively low availability (Zhao et al., 2022)*.* Although LCS has long been used as a prescription to treat liver injuries, its underlying mechanisms remain unclear.

In our previous study, the pharmacological effects of LCS were investigated in treating liver injuries induced by CCl_4_ and hepatitis B caused by HBV, *in vivo* and *in vitro* (Ren et al., 2019; Zhao et al., 2019). In the present study, network pharmacology was used to dissect the key active ingredients and pharmacological mechanisms of LCS against liver injuries by analyzing the interactions among candidate compounds, potential targets, and relevant pathways. Then the metabonomics method was used to explore the intervention mechanism of LCS in the treatment of liver injuries. Based on UPLC/Q-TOF-MS, the metabonomics assay was conducted on a pathological model of CCl_4_-induced rat liver injury model combined with multivariate data analysis. Furthermore, the crucial targets of the shared pathways of network pharmacology and metabonomics were experimentally validated to account for the therapeutic effects of LCS in liver injury.

## 2 Materials and Methods

### 2.1 Plant Materials and Preparation of the Aqueous Extracts of Le-Cao-Shi

The whole plant of *A. ilicifolius* was collected from Jiangmen, Guangdong Province, China. The aerial parts of *P. pulchellum* were from Meizhou, Guangdong Province, China, and the radixes of *C. cochinchinensis* were from Hechi, Guangxi Province, China. The aforementioned plants were botanically authenticated by Professor Feng-Qin Zhou (from Shandong University of Chinese Medicine). These herbs were shade dried, weighted, powered, mixed in proportion (*A. ilicifolius*, 200 g; *P. pulchellum*, 80 g; *C. cochinchinensis*, 120 g), and immersed in 4,000 ml of water (w/v, 1:10) for 1 h, then heated to reflux at 100°C for 2 h. The extraction solution was filtered through six-layer gauze, and the residue was refluxed again in 3,200 ml of water (w/v, 1:8) at 100°C for 1.5 h. All of the filtrates were combined and volatilized to dryness by using a rotary evaporator (Heidolph, Germany) at 45°C to obtain an aqueous extract.

### 2.2 Chemicals and Reagents

Chemical carbon tetrachloride (CCl_4_) was purchased from Tianjin Tianli Chemical Reagent Co., Ltd. (Tianjin, China). Silymarin was obtained from Beijing Solarbio Science & Technology Co., Ltd. (Beijing, China). The diagnostic kits specific for necrosis factor-α (TNF-α), interleukin-6 (IL-6), interleukin-1β (IL-1β), superoxide dismutase (SOD), catalase (CAT), glutathione peroxidase (GSH-Px), and malondialdehyde (MDA) were obtained from the Jiancheng Institute of Biotechnology (Nanjing, China). Chromatography-grade methanol, acetonitrile, ammonium hydroxide, and ammonium acetate were provided by CNW Technologies (Shanghai, China). All of the other chemicals and reagents used in the experiments were of analytical grade.

### 2.3 Animals and Treatments

Sprague Dawley rats (200 ± 20 g) were supplied by Beijing Vital River Laboratory Animal Technology Co., Ltd. All rats were kept in-house under the following conditions: 20 ± 2°C temperature, 60–70% relative humidity, and 12-h light/dark cycle and were fed with standard food and water *ad libitum*. All of the experimental animals were treated in accordance with the guidelines of the Chinese Council for Animal Care.

After an environmental adaptation period of 7 days, rats were randomized into six groups (*n* = 8), including normal control group, model group (CCl_4_-treated), positive control group, high-dose LCS treatment group (LCS-H), middle-dose LCS treatment group (LCS-M), and low-dose LCS treatment group (LCS-L). The rats in the model and normal control groups were administered by gavage with normal saline (1 ml/kg/d), the rats in the positive control group were with silymarin (100 mg/kg/d), and LCS-H, LCS-M, and LCS-L groups were with 12, 6, and 3 g crude herbs/kg/d of LCS, respectively. As for the dosage of LCS groups, it was recorded that the adult daily dose was 60 g/60 kg in Chinese Marine Materia Medica (Guan and Wang, 2009). Animal dosages were inferred according to the following formula: adult daily dose of 60 g × 0.018/200 × 1,000×the multiple of the clinical equivalency dose (Shi et al., 2014). All of the drug treatments were maintained for 27 continuous days. On the 28th day, the rats in the normal control group were intraperitoneally injected with 1 ml of olive oil, while in the other groups, they were given the same volume of 50% CCl_4_ olive oil mixture (v/v) for acute liver injury modeling. Finally, all of the rats were killed after fasting while being given enough water for 24 h. Blood samples were taken from the eye socket and into 1.5-ml Eppendorf (EP) tubes, followed by centrifugation at 3,000 *rpm* for 10 min. The supernatant was transferred into another 1.5-ml EP tube and stored at 4°C until use. The rat livers were excised and kept at ‒80°C.

### 2.4 Network Pharmacology Analysis

Network pharmacology analysis procedures were summarized in Supplementary Figure S1. Ingredients from the three single herbs of LCS were collected from the literature. Candidate compounds were screened based on ADME parameters, and potential targets and pathways involved in liver injuries were predicted based on compound–target online databases. Through network construction and contribution index (CI) calculation, the active compounds in LCS were determined.

#### 2.4.1 Chemical Ingredient Database Construction

Compounds in the three single herbs of LCS were collected from the Chemical Database of the Academy of Sciences (http://www.organchem.csdb.cn/scdb/default.asp), and manually supplemented through text mining (Yue et al., 2017; Zhao et al., 2022). Their chemical structures were obtained from the Chemical Book database (http://www.chemicalbook.com), NCBI PubChem database (http://www.ncbi.nlm.nih.gov/pccompound), and the literature, and drawn by using Chemdraw software (https://www.perkinelmer.com.cn/category/chemdraw, version 3.7.2). Compound formats were saved as mol2 and SMILES, respectively, for further analysis.

#### 2.4.2 Candidate Compound Screening

Based on the TCMSP database (https://lsp.nwu.edu.cn/gj.htm), ADME parameters such as oral bioavailability (OB) value, Caco-2 permeability, and drug-like index (DL) could be obtained (Ru et al., 2014). The screening thresholds were set as OB ≥ 30%, Caco-2 ≥ −0.14, and DL ≥ 0.1 (Li et al., 2016; Yue et al., 2017). Compounds containing glycosyl groups were further deglycosylated and expressed as compound_qt (Zhao et al., 2022).

#### 2.4.3 Target Prediction

Targets of candidate compounds were searched in the Drug Bank database, Similarity Ensemble Approach (SEA) database, STITCH database, and a reverse pharmacophore-based screening platform, Pharm Mapper database (Knox et al., 2011; Kuhn et al., 2014; Liu et al., 2015; Wang et al., 2017; Xu et al., 2018; Zhao et al., 2022). These predicted targets were chosen by the rank of matching score and then input into the Therapeutic Target Database (TTD), Online Mendelian Inheritance in Man (OMIM) database, Drug Bank database, and PharmGKB database (Altman, 2007; Wishart et al., 2008; Jiang et al., 2015; Liu et al., 2015). Combined with the literature, targets related to liver injuries as potential targets for next analysis were retained.

#### 2.4.4 Gene Ontology and KEGG Pathway Enrichment Analysis

Database for Annotation, Visualization, and Integrated Discovery (DAVID) was used to perform Gene Ontology (GO) enrichment analysis of the potential targets of candidate compounds in LCS. Pathway enrichment was analyzed using pathway data associated with liver injuries obtained from the Kyoto Encyclopedia of Genes and Genomes (KEGG) pathway section (Kanehisa and Goto, 2000; Tang et al., 2016).

#### 2.4.5 Network Construction and Analysis

The candidate compound–potential target (cC-pT) network and potential target–pathway (pT-P) network were established by using Cytoscape (version 3.7.2), open software package for visualizing and analyzing interaction networks, to generate a bipartite network (Smoot et al., 2011).

#### 2.4.6 Contribution Index Calculation

The contribution index (CI) based on network-based efficacy (NE) and the literature was calculated to estimate the contribution of each candidate compound to the anti-liver injury effects of LCS by the following equations:

$$NE\left(j\right)=\sum_{i=1}^{n}d_{i},$$ (1)

$$CI\left(j\right)=\frac{c_{j}\times NE\left(j\right)}{\sum_{i=1}^{m}c_{i}\times NE\left(i\right)}\times 100\%,$$ (2)

where *n* is the number of targets associated with ingredient *j* in the cC-pT network, *d*_*i*_ is the degree of target *i* associated with ingredient *j* in the pT-P network, *c*_*i*_ is the number of anti-liver injury-related literature of ingredient *i*, and *m* is the number of ingredients. For literature mining, liver injury together with the name of each candidate compound was used as keywords for information retrieval. Articles published in 1995–2022 were obtained.

#### 2.4.7 Molecular Docking

Molecular docking was performed by using MOE software (Molecular Operating Environment, version 2020) to evaluate the interaction between targets and candidate compounds. To be specific, the X-ray crystal structures of target proteins were downloaded from the Protein Data Bank (www.rcsb.org/). The energy of the enzyme was minimized by deleting the water molecules and the ligands used for crystallization. The 2D structures of candidate compounds (ligand molecules) were drawn in ChemDraw software (version 15.0) and saved in the 3D SDF format. The database was created by transferring it to the MOE system, and then saved as an .mdb file extension. Then the surface of the macromolecule was scanned, and optimization processes were performed to find the active sites on the enzyme. Through generating 30 most stable conformers and electrostatic and van der Waals interactions, the grid scores were calculated to obtain the optimal spatial conformations. All parameters were set to default. Fit values were obtained and ranked to evaluate the possibility of binding of candidate compounds to targets.

### 2.5 Metabonomics Analysis

#### 2.5.1 Sample Collection and Preparation

Liver tissue samples from normal control, model, and high-dose LCS groups were homogenized in ninefold cold normal saline (w/v). The liver samples (each weighing 50 mg) were deproteinized with 1,000 μL methanol and vortexed for 30 s. Then the mixed solutions were centrifuged at 12,000 *rpm* at 4°C for 15 min. Afterward, supernatants (each 500 μL) were transferred into new EP tubes, and they were evaporated to dryness at 37°C using a vacuum concentrator. The residues were re-dissolved with 100 μL of extraction solution acetonitrile–water (1:1, v/v), vortexed for 3 min, and centrifuged at 12,000 *rpm* (4°C) for 15 min. Meanwhile, a quality control pooled (QCP) sample was prepared by mixing 10 μL of each test sample. The supernatants were filtered through a syringe filter (0.22 μm) and transferred into an autosampler vial for the following UPLC-Q-TOF/MS analysis. During the experimental period, all samples were kept at ‒80°C until used.

#### 2.5.2 UPLC-Q-TOF/MS Analysis

An Agilent Acquity UPLC system (Agilent Technologies Inc., California, United States) was interfaced with a Triple TOF MS system (AB Sciex, Massachusetts, United States) equipped with an electrospray ionization (ESI) source. A 2 μL aliquot of each vial was injected into an Acquity UPLC@BEH C_18_ column (1.7 μm, 2.1 × 100 mm) for separating complex components. The column temperature was maintained at 30°C. The mobile phase, which consisted of water with 25 mM NH_4_Ac and 25 mM NH_4_OH (pH = 9.75) (A) and acetonitrile (B), was pumped at a flow rate of 0.5 ml/min under the following elution program: 0–0.5 min, 5% B; 0.5–7 min, 5%–35% B; 7–8 min, 35%–60% B; 8–9 min, 60%–60% B; 9–9.1 min, 60%–5% B; 9.1–13 min, and 5%–5% B. The MS parameters were as follows: collision energy spread, 30 eV; ion spray voltage, 5.0 kV (+) and 4.0 kV (−); capillary and heater temperature, both 650°C; and sheath and auxiliary gas flow rate, 35 and 60 psi, respectively.

#### 2.5.3 Data Processing and Multivariate Data Analysis

The acquired MS raw data were converted to the mzXML format using ProteoWizard software (version 3.2) and imported to XCMS software (version 3.2) for automatic data processing, including peak extract, peak alignment, and peak picking, to produce peak data matrix containing retention time (*R*_t_), *m*/*z* data, and peak intensity. Then, the resultant data matrices were further imported to SIMCA software (version 14.1) for multivariate data analysis, including principal component analysis (PCA) and orthogonal partial least squares discriminant analysis (OPLS-DA). PCA was employed to visualize the clustering, trends, and outliers among samples, and OPLS-DA was used to identify the differential metabolites that were responsible for the difference between two groups (Wu et al., 2015; Wu et al., 2017; Yang et al., 2017a).

#### 2.5.4 Potential Biomarker Identification and Metabolic Pathway Analysis

OPLS-DA was employed to identify differential variables according to their VIP (variable influence on projection) values. Variables with VIP value >1 were selected and further input into Student’s *t*-test to determine the significance of each variable. Variables with VIP >1 and *p* < 0.05 were selected as candidate biomarkers. Candidate biomarkers were identified by automated comparison of retention time (*R*_t_)-*m*/*z* datasets to a commercially available metabonomics library (provided by Biotree Biotech, Shanghai, China). Metabolic pathway analysis was conducted with MetaboAnalyst 4.0 based on the KEGG database to reveal the crucial metabolic pathways (Kanehisa et al., 2014; Chong et al., 2018).

### 2.6 Experimental Validation

#### 2.6.1 Serum Inflammatory Biomarker-Level Determination

Serum TNF-α, IL-6, IL-1β, and NO levels were measured by commercial ELISA kits according to the manufacturer’s instructions.

#### 2.6.2 Hepatic Antioxidant Enzyme Activities and Lipid Peroxidation Degree Determination

Each supernatant sample from the homogenized liver tissue was used to measure the activities of hepatic antioxidant enzyme SOD, CAT, and GSH-Px, and the lipid peroxidation product MDA level by using commercially available diagnostic kits according to the manufacturer’s instructions.

#### 2.6.3 Statistical Analyses

The data were expressed as means ± standard deviation (SD). One-way analysis of variance and Student’s *t*-test were used to analyze the differences between groups, and *p* < 0.05 was considered statistically significant, while *p* < 0.01 was considered highly significant.

## 3 Results and Discussion

### 3.1 Network Pharmacology Analysis

#### 3.1.1 Construction of Chemical Ingredient Database

Ingredients in the three single herbs of LCS were manually collected, including 126 ingredients in *A. ilicifolius* (see Supplementary Figure S2), 50 in *P. pulchellum* (see Supplementary Figure S3), and 43 in *C. cochinchinensis* (see Supplementary Figure S4). After removing duplication, the total number of compounds was 213.

#### 3.1.2 Selection of Candidate Compounds

TCM prescriptions are usually orally administered, and only when absorbed in blood can compounds show bioactivity (Wang et al., 2011). Oral bioavailability (OB), as one of the most important pharmacokinetic parameters, determines whether a constituent is pharmaceutically active. Compounds with adequate Caco-2 permeability and drug-likeness (DL) often exhibit pharmacodynamic action on target sites (Luo et al., 2016). Moreover, a few active compounds that do not meet all the three criteria were selected for their high amounts and high bioactivities. For example, oleanolic acid (AI01) has a relatively low OB value (<30%), whereas it is a hepatoprotective drug, so it was counted in for further analysis (Zhu et al., 2019). Similarly, other seven compounds were also regarded as candidate components, including (‒)-epicatechin (PP10), (‒)-gallocatechin (PP11), and (‒)-epigallocatechin (PP12), reported with hepatoprotective activities on a cellular level, acteoside (AI89), isoacteoside (AI90), and 4-hydroxy-2(3H)-benzoxazolone (AI37) with anti-liver injury effects, and adenosine (AI53) with anti-inflammatory effects (Cronstein, 1994; Xiong et al., 1998; Kanchanapoom et al., 2001; Liu et al., 2013; Ma et al., 2015; Saranya et al., 2015; Fan et al., 2018). It should be pointed out that five compounds were not available in the TCMSP database, including blepharigenin (AI41), (2*R*)-2-*O*-β-*D*-glucopyranosyl-2*H*-1,4-benzoxazin-3(4*H*)-one (AI42), (2*R*)-2-*O*-*b*-*d*-glucopyranosyl-5-hydroxy-2*H*-1,4-benzoxazin-3(4*H*)-one (AI45), 7-Cl-(2*R*)-2-*O*-*β*-*D*-glucopyranosyl-2*H*-1,4-benzoxazin-3(4*H*)-one (AI47), and 2,6-dimethoxy-*p*-hydroquinone-1-*O*-*β*-*D*-glucopyranoside (AI71), whereas they were frequently reported with significant anti-liver injury effects; thus, they were also reserved as candidate compounds (Kanchanapoom et al., 2001; Huo et al., 2005; Lin et al., 2013; Saranya et al., 2015). Consequently, 57 candidate compounds were selected from the 213 compounds in LCS (ADME parameters of 57 candidate compounds see Supplementary Table S1), with 39 from *A. ilicifolius*, 17 from *P. pulchellum*, and 6 from *C. cochinchinensis*.

#### 3.1.3 Potential Targets Associated With Liver Injuries in LCS

Among 57 candidate compounds, only 7 candidate compounds, AI12, AI41, AI49, AI53, AI55, AI71, and AI110, related with no target associated with liver injuries, and the other 50 compounds yielded 87 potential targets. (For information of 87 potential targets, see Supplementary Table S2.)

#### 3.1.4 Gene Ontology Enrichment Analysis

**FIGURE 1:**
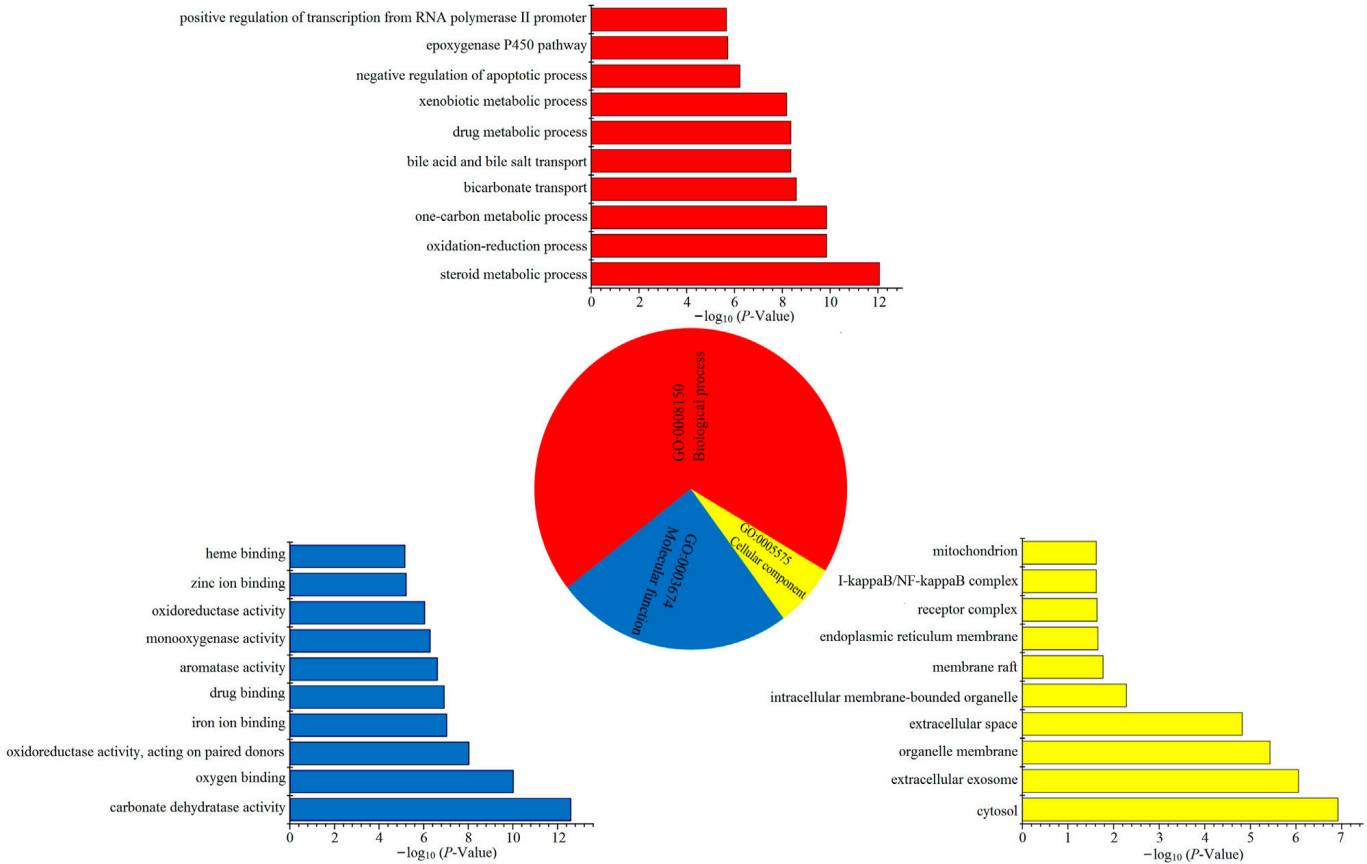
GO enrichment analysis of the potential targets based on the active ingredients from LCS.

The platform DAVID provides a comprehensive set of functional annotation tools to understand the biological meaning behind a large list of genes (Huang et al., 2008; Xu et al., 2018). The Gene Ontology (GO) annotation system in DAVID could be used to assign functions to genes (Li et al., 2015). The GO annotation system contains three independent categories: biological processes (BP), molecular function (MF), and cellular components (CC). In the present study, three categories in GO annotation, namely, BP, MF, and CC, accounted for 69.04, 24.55, and 6.41%, respectively, with the top 10 significantly enriched terms in BP, MF, and CC categories, as shown in Figure 1. The key BPs were steroid metabolic process, oxidation–reduction process, bile acid and bile salt transport, drug metabolic process, xenobiotic metabolic process, and negative regulation of apoptotic processes, indicating that LCS could mainly regulate inflammatory, oxidation–reduction, and bile transport processes. The key MFs were carbonate dehydratase activity, oxygen binding, oxidoreductase activity, iron ion binding, drug binding, aromatase activity, monooxygenase activity, zinc ion binding, and heme binding, mainly related to the redox system. As for CC categories, most of the predicted targets were located in the cytoplasmic part, extracellular region, organelle membrane, and organelle, which indicated that the anti-liver injury effects of LCS could be implemented through the processes of preserving cellular structural integrity, repairing hepatic tissue damage, and regulating hepatic cellular homeostasis.

#### 3.1.5 Network Construction and Analysis

In this study, two visualized networks were constructed, namely, candidate compound–potential target and potential target–pathway networks, which could help decipher the relationships among active compounds, targets, and pathways.

##### 3.1.5.1 Candidate Compound–Potential Target Network

**FIGURE 2:**
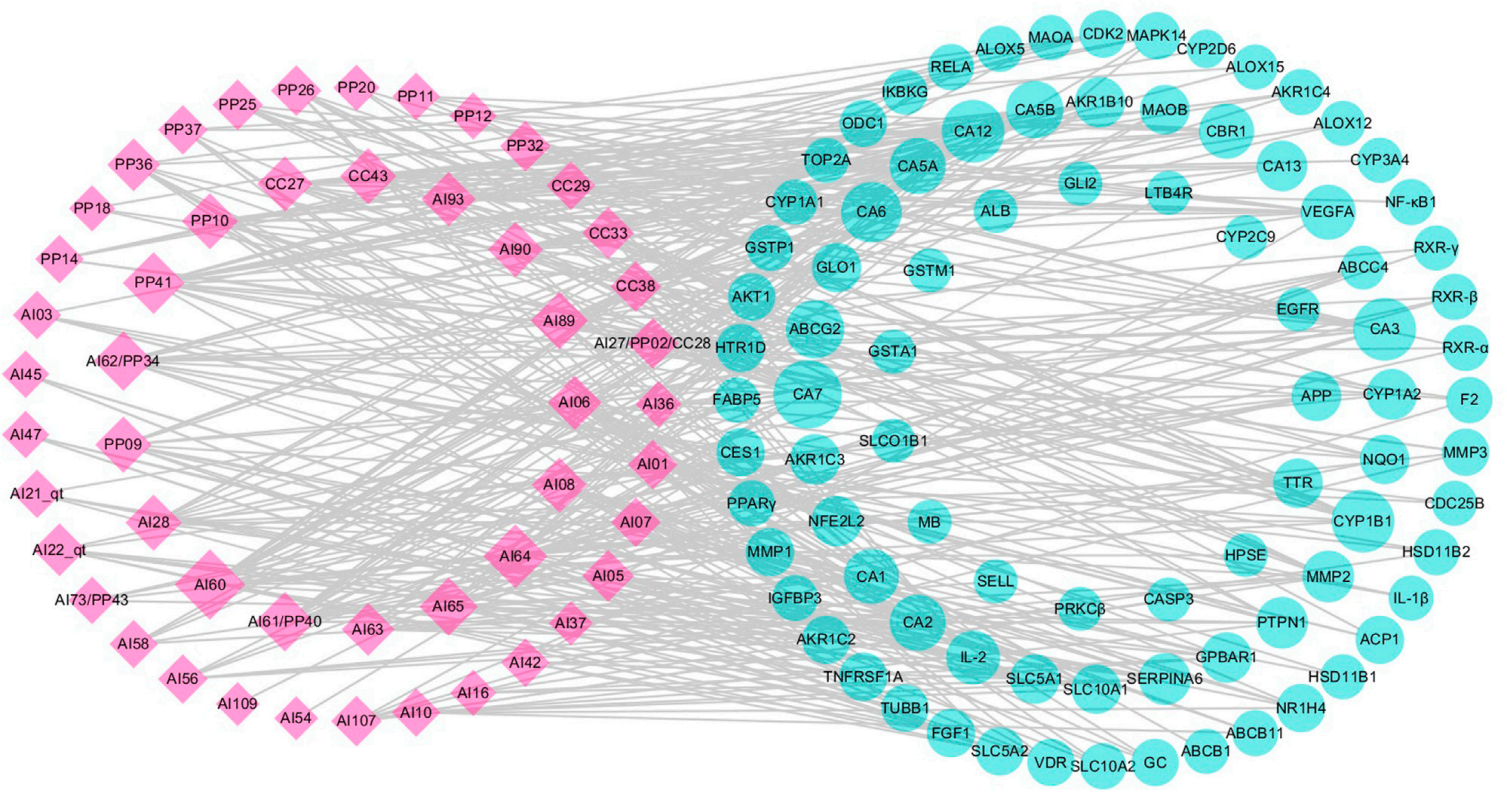
Candidate compound–potential target (cC-pT) network associated with liver injury based on the active ingredients from LCS. Note: the pink diamond and cyan circle nodes represent candidate compounds and potential targets, respectively.

To visualize the complex interactions between candidate compounds in LCS and their potential targets, a bipartite graph of the cC-pT network was integrated by connecting 50 candidate compounds and 87 potential targets (Figure 2). In this network, pink diamond nodes represent candidate compounds and cyan circle nodes represent potential targets. The node degree is the interaction number of a node, and node size is appropriate to node degree.

Among the candidate compounds, 12 compounds were found to have a high node degree, hitting more than 10 potential targets. For example, ferulic acid (AI60) had the highest node degree (degree = 25), followed by vanillic acid (AI64, degree = 19), caffeic acid ester (PP41, degree = 17), p-hydroxybenzoic acid (AI62/PP34, degree = 14), syringic acid (AI65, degree = 13), and acteoside (AI89, degree = 13). Compounds, such as bergapten (CC43), (+)-catechin (PP09), (‒)-epicatechin (PP10), and luteolin (AI28), were with 12 potential targets, isoacteoside (AI90) and *β*-sitosterol (AI08) with 11 potential targets, and cholesterol (AI06) with 10 potential targets. These compounds with high node degrees are likely to play a vital role in treating liver injury.

The 87 potential targets were relevant to pathogenic processes of liver injuries, mainly including xenobiotic metabolism by cytochrome P450 enzymes, inflammatory response, lipid peroxidation, bile metabolism, and apoptosis. Among them, 14 targets were connected with more than 7 candidate compounds, including carbonic anhydrases (CAs), cytochrome P450 1B1 (CYP1B1), ATP-binding cassette subfamily G member 2 (ABCG2), vascular endothelial growth factor A (VEGFA), carbonyl reductase (NADPH) 1 (CBR1), interleukin-2 (IL-2), tyrosine-protein phosphatase non-receptor type 1 (PTPN1), corticosteroid-binding globulin (SERPINA6), matrix metalloproteinase 2 (MMP2), aldo-keto reductase family 1 member B10 (AKR1B10), aldo-keto reductase family 1 member C3 (AKR1C3), cytochrome P450 1A2 (CYP1A2), G-protein-coupled bile acid receptor 1 (GPBAR1), and nuclear factor erythroid 2-related factor 2 (NFE2L2).

Target CA2 is a zinc-binding protein associated with hepatitis, and CA3 is expressed in the epithelial cells of the bile ducts involved in many liver diseases, including chronic viral hepatitis and primary biliary cirrhosis (Sly and Hu, 1995; Suzuki et al., 1999; Nishimori et al., 2004). Inflammatory cytokines, such as interleukin-1β (IL-1β), nuclear factor-kappa B (NF-κB1), and leukotriene B4 receptor 1 (LTB4R), could be secreted by activated inflammatory cells and initiate a series of signaling pathways, resulting in damage to tissues (Gu et al., 2013; Schroen et al., 2015; Tang et al., 2016; Yang et al., 2017b). Inflammatory cytokines could also enhance matrix metalloproteinase (MMP) expression, such as MMP1, MMP2, and MMP3. These enhanced MMPs could then decrease proteoglycans and native collagen and lead to further inflammatory responses (Goldring, 2000; Rotte et al., 2012; Tao et al., 2015). Mitogen-activated protein kinase 14 (MAPK14) and tumor necrosis factor receptor superfamily member 1A (TNFRSF1A) are involved in the inflammation response through the MAPK signaling pathway (Ji et al., 2015; Luo et al., 2016). Arachidonate 5-lipoxygenase (ALOX5) and AKR1C3 could achieve their anti-inflammatory activities by suppressing lipid metabolism and the production of pro-inflammatory cytokines in the arachidonic acid metabolic pathway. Peroxisome proliferator-activated receptors (PPARs) are members of the nuclear hormone receptor superfamily, among which PPARγ is strongly linked to inflammatory response (Afif et al., 2007; Chang et al., 2012). Cytochrome P450 enzymes, such as CYP2E1, CYP2C19, and CYP7A1, are related to lipid homeostasis and xenobiotic metabolism (Girish et al., 2009; Zhang Y. et al., 2014; Haiyu et al., 2015; Chen et al., 2016). For instance, CYP7A1 could maintain cholesterol homeostasis by encoding the rate-limiting enzyme that catalyzes the conversion of cholesterol into bile acids, while CYP2E1 could metabolize xenobiotics (Girish et al., 2009; Chen et al., 2016). Cyclin-dependent kinase (CDK2), vitamin D nuclear receptor (VDR), transcription factor p65 (RELA), and VEGFA are related to cell proliferation and apoptosis (Zhang Y. et al., 2014; Ji et al., 2015; Christian et al., 2016; Zheng et al., 2017). ATP-binding cassette subfamily B member 11 (ABCB11), also known as the bile salt export pump (BSEP), is a dominant efflux system associated with cholestatic liver injury (Stieger et al., 2007; Girish et al., 2009; Woodhead et al., 2014). Information of the aforementioned targets indicated that LCS could achieve its anti-liver injury effects through xenobiotic metabolism by cytochrome P450 enzymes, suppressing inflammatory response, lipid peroxidation, and apoptosis, and regulating bile metabolism.

##### 3.1.5.2 Potential Target–Pathway Network

**FIGURE 3:**
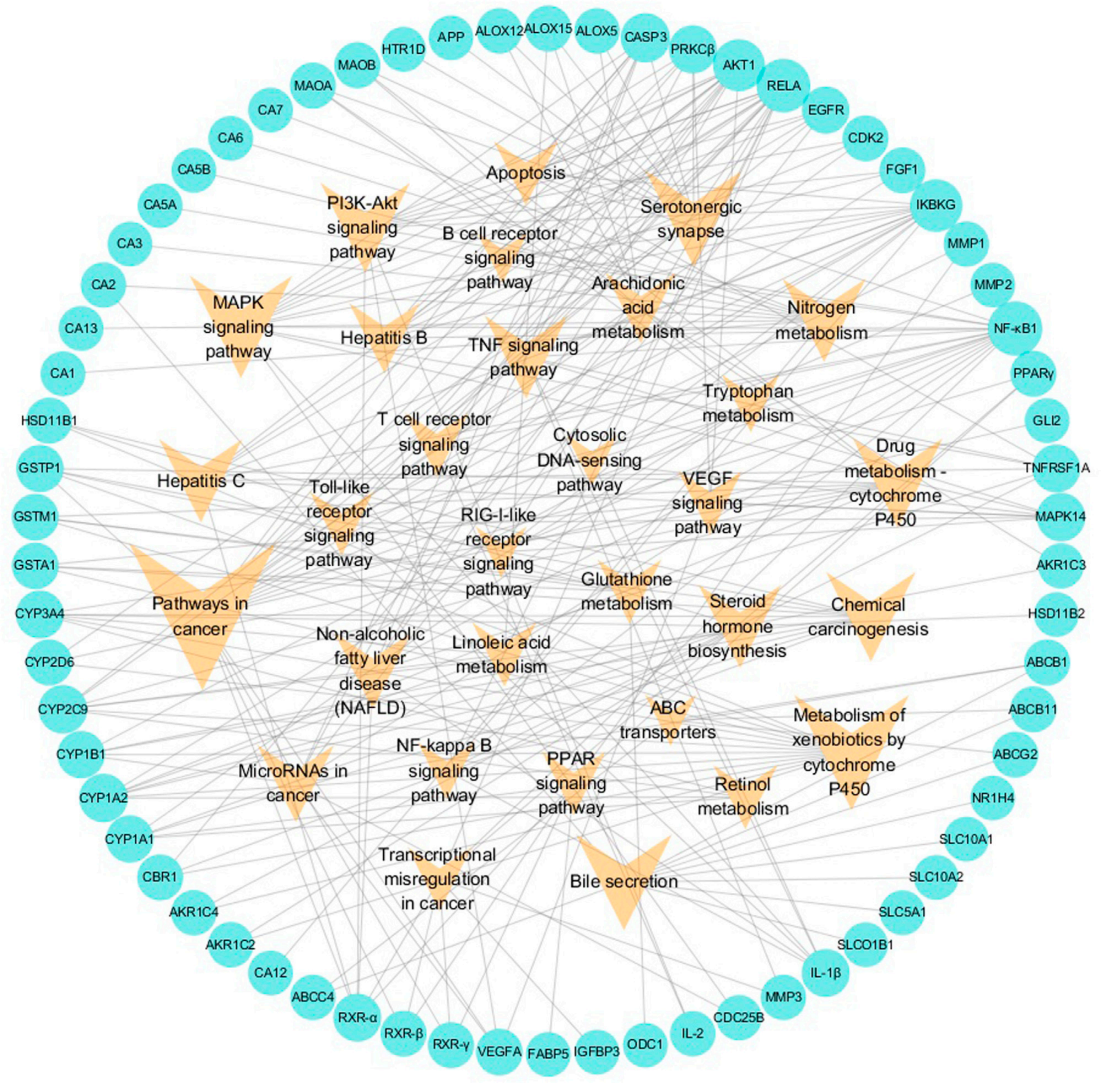
Potential target–pathway (pT-P) network associated with liver injury based on the active ingredients of LCS. Note: the cyan circle and V-shaped nodes represent potential targets and pathways, respectively.

In the pT-P network, 69 potential targets and 31 pathways related to liver injuries were mapped (Figure 3). The pathways were extracted from the KEGG database, a collection of pathway maps representing information on molecular interactions (Kanehisa and Goto, 2000; Tang et al., 2016). Target NF-κB1 had the highest degree of 16. RELA, NF-κB essential modulator (IKBKG) and RAC-α serine/threonine-protein kinase (AKT1) had the degrees of 15, 13 and 12, respectively. Caspase-3 (CASP3), MAPK14, CYP1A2, CYP2C9, CYP3A4, protein kinase C beta type (PRKCβ), TNFRSF1A, IL-1β, and retinoic acid receptor RXR-α had degrees of above 6. Among these targets, RELA was reported to be associated with cell cycle, proliferation, and cell death (Christian et al., 2016). Inflammatory cytokines, such as IL-1β and NF-κB1, are significant in strengthening inflammatory reactions (Dinarello, 2009; Santangelo et al., 2012).

Among the 31 pathways, there were more than 15 that could be mainly divided into cancer, xenobiotic metabolism by cytochrome P450, bile metabolism, inflammation, and antioxidation modules. Among them, pathways involving cancer, chemical carcinogenesis, metabolism of xenobiotics by cytochrome P450, drug metabolism-cytochrome P450, bile secretion, MAPK signaling, and PI3K-Akt signaling hit more than 10 potential targets, and the glutathione metabolism pathway hit 3 potential targets. The pathway in cancer had the highest node degree in the pT-P network. Liver cancer often results from underlying liver injuries and is mostly associated with hepatitis and cirrhosis (Thorgeirsson and Grisham, 2002). The VEGF signaling pathway is an extensively studied pathway in blocking tumor angiogenesis (Sharan and Woo, 2015). Cytochrome P450 superfamily isoforms could metabolize xenobiotic and drug (Chen et al., 2016). Two cytochrome P450 pathways, metabolism of xenobiotics by cytochrome P450 and drug metabolism-cytochrome P450, were important pathways in the pT-P network. Many compounds were reported to regulate hepatic cytochrome P450 enzymes to protect the liver against xenobiotics *via* antioxidants and the suppression of glutathione depletion and electrophilic radical formation (Sugiyama et al., 2006; Girish et al., 2009). Drug metabolism in the liver could be realized through the enhancement of drug-metabolizing cytochrome P450 enzyme activity (Girish et al., 2009; Ji et al., 2015). Dysfunctions of bile formation, secretion, and transportation are the main biological factors contributing to cholestatic liver injury (Stieger, 2011). Bile is a complex aqueous secretion consisting of about 95% water and 5% organic and inorganic solutes, such as bile salt, phospholipid, protein, cholesterol, and bilirubin (Boyer, 2013). Bile salt, the major organic solute in bile, is synthesized from cholesterol in hepatocytes to keep cholesterol homeostasis (Reshetnyak, 2013). In the pT-P network, target ABCB11 (or BSEP), an acknowledged dominant efflux system associated with cholestatic liver injury, was related to the bile secretion pathway.

**FIGURE 4:**
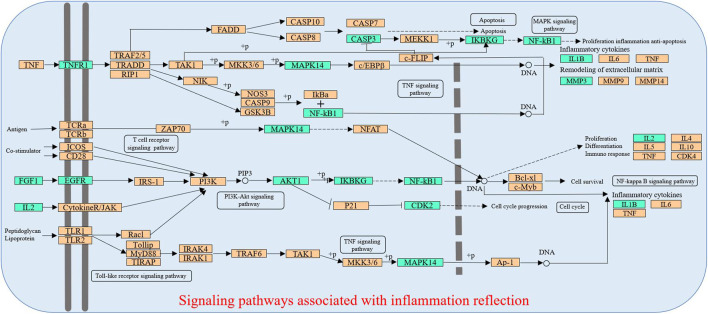
Distribution of partial targets of LCS on the compressed pathways. Note: The cyan nodes are potential targets of LCS, while the orange nodes are relevant targets in the pathway.

In the pT-P network, the MAPK signaling pathway, PI3K-Akt signaling pathway, TNF signaling pathway, arachidonic acid metabolism pathway, T-cell receptor signaling pathway, toll-like receptor signaling pathway, NF-kappa B signaling pathway, and linoleic acid metabolism pathway were involved in the inflammatory process. The PI3K-Akt signaling pathway could suppress inflammation (Schabbauer et al., 2004). The arachidonic acid metabolism pathway was the upstream pathway in many inflammation reflections (Gu et al., 2013; Tufvesson et al., 2015). The NF-kappa B pathway was important in anti-inflammatory process, and its relevant inflammatory mediators mainly include tumor necrosis factor-α (TNF-α) and IL-1β (Sen and Smale, 2010). TNF-α could promote the production of other inflammatory cytokines such as IL-1 (Balakumar and Singh, 2006). The linoleic acid metabolism pathway was reported relevant to inflammatory processes (Tufvesson et al., 2015). Liver injury could cause a serious inflammatory reaction in the liver, so the regulation of inflammation is important. The compressed inflammatory pathway extracted from the KEGG database (Figure 4) indicated that LCS could synergistically act on multiple inflammatory targets such as NF-κB1, AKT1, IKBKG, MAPK14, IL-1β, and IL-2 in many pathways, mainly including the T-cell receptor signaling pathway, toll-like receptor signaling pathway, PI3K-Akt signaling pathway, TNF signaling pathway, NF-kappa B signaling pathway, and MAPK signaling pathway.

In addition, when xenobiotics bind to proteins, lipids, or nuclei, oxidative stress can be generated in the human body, mainly including the depletion of reduced glutathione, the inducement of lipid peroxidation, and the generation of free radicals (Maddrey, 2005). These oxidative stresses could affect mitochondrial function and inhibit the movement of calcium from the cytosol, causing the death of hepatocytes (Rang et al., 2003). Therefore, it is supposed that LCS could inhibit liver injuries by mediating the glutathione metabolism pathway to suppress lipid peroxidation and improve antioxidant defense system.

In summary, the anti-liver injury effects of LCS could be implemented by mediating a group of pathways relevant to cancer, xenobiotics, metabolism by cytochrome P450, bile metabolism, inflammation, and antioxidation.

#### 3.1.6 Contribution Index Analysis

**FIGURE 5:**
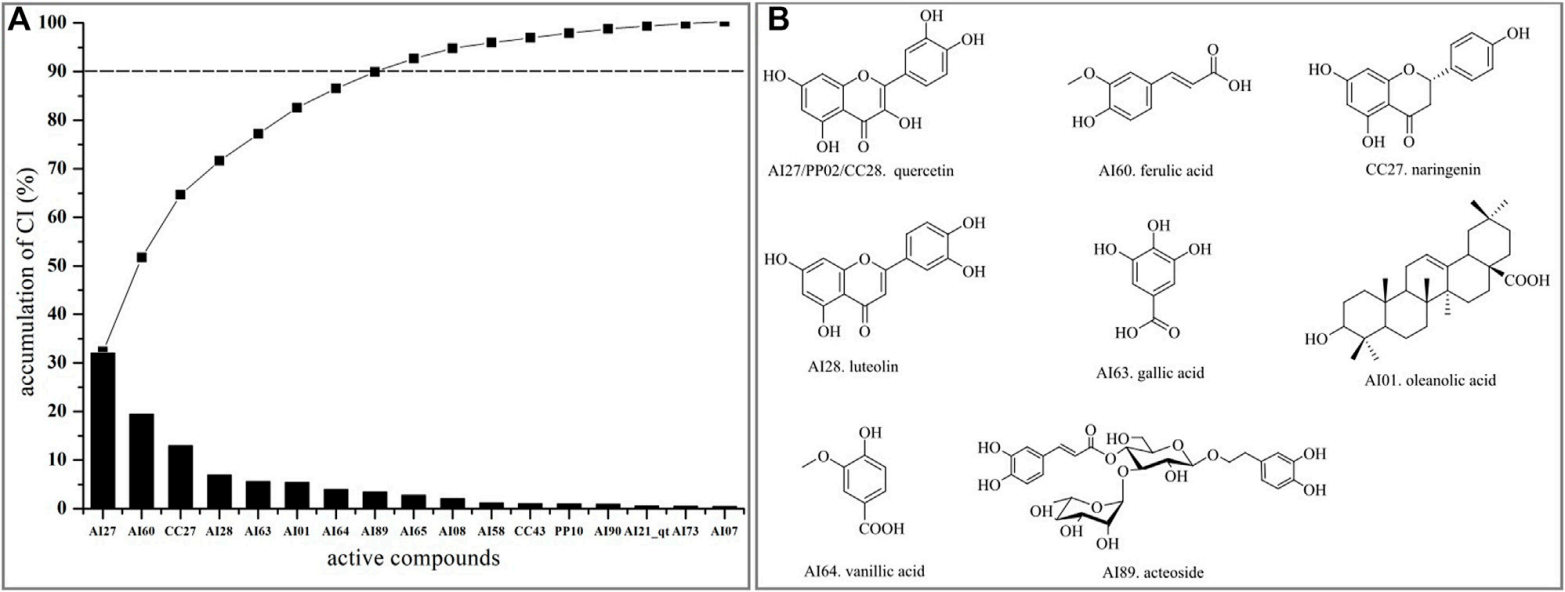
CI value and active compounds in LCS. **(A)** CI value and accumulative CI of active compounds in LCS; **(B)** active compounds in LCS with accumulative CIs of more than 90%.

Integrating with the aforementioned two networks, the CI of every candidate compound was calculated based on NE and weighted by the literature. According to CI calculated results (Figure 5A, Supplementary Table S3), eight active compounds displayed the most contribution to the anti-liver injury effects of LCS with a sum of CIs of more than 90%, namely, quercetin (AI27/PP02/CC28), ferulic acid (AI60), naringenin (CC27), luteolin (AI28), gallic acid (AI63), oleanolic acid (AI01), vanillic acid (AI64), and acteoside (AI89) (Figure 5B).

Quercetin (AI27/PP02/CC28) was reported to have radical-scavenging activities and inhibitory activities on lipopolysaccharide-induced inflammatory both *in vitro* and *in vivo* (Shen et al., 2002; He et al., 2011). Ferulic acid (AI60) had the ability of inhibiting lipid peroxidation, antioxidation, and anti-inflammatory by reducing the expression of TNF-α and IL-1β in lipopolysaccharide-activated macrophages (Xu et al., 2013; Navarrete et al., 2015). Naringenin (CC27) could attenuate CCl_4_-induced hepatic inflammation by activating the Nrf2-mediated pathway in rats (Esmaeili and Alilou, 2014). Luteolin (AI28) could exert anti-inflammatory effects by preventing arachidonic acid synthesis and scavenging hydrogen peroxide and had antioxidant and anticancer properties (Odontuya et al., 2005; Lin et al., 2008). Gallic acid (AI63) could inhibit pro-inflammatory cytokine production and histamine release in mast cells (Kim et al., 2006). Oleanolic acid (AI01) could protect the liver against many hepatotoxicants (Liu et al., 2008). Vanillic acid (AI64) could exert anti-inflammatory activity by regulating the expression of pro-inflammatory cytokines (Ziadlou et al., 2020). Acteoside (AI89) could modulate inflammatory responses through the NFκβ/Iκβ signaling pathway in alcohol-induced hepatic damage and possess many other pharmacological activities, such as antioxidant and antitumor activities, and hepatoprotective effects against CCl_4_-induced liver injury (Wong et al., 2001; Khullar et al., 2019; Zhang et al., 2020). The aforementioned summary of the eight key active compounds in LCS further indicated that the anti-liver injury mechanism of LCS was largely related to anti-inflammatory and antioxidant activities.

#### 3.1.7 Molecular Docking

**FIGURE 6:**
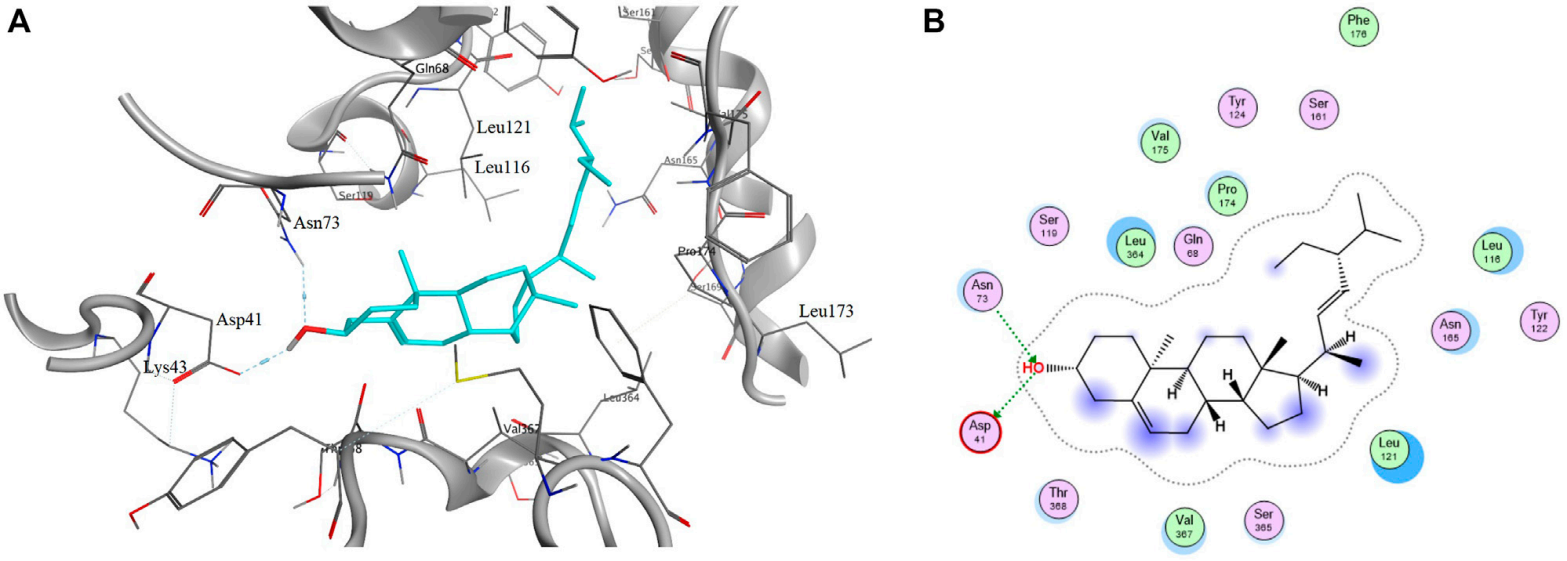
Binding mode of protein 6QD5 with molecule AI07 from LCS. Note: A and B are the 2D and 3D binding mode, respectively.

Molecular docking was used to evaluate the interactions between candidate compounds in LCS and their potential targets. The molecular docking results of 40 representative targets are listed in Supplementary Table S4. According to the molecular docking results of 40 representative targets and their corresponding compounds, it was suggested that these potential targets associated with liver injuries could finely bind to their connected compounds. Target SLC10A1 that is bile acid cotransporter was taken as an example. The 6QD5 protein of target SLC10A1 had the best binding conformation with AI07 (grid score = ‒8.49016). Their binding mode showed that AI07 was well accommodated inside the binding pocket of 6QD5, mainly through hydrogen bond interaction, and the main binding amino acid residues were Asn73 and Asp41 (Figure 6).

### 3.2 Metabonomics Analysis

To investigate the alterations of rat liver metabonomics after administration of formula LCS, an UPLC-Q-TOF/MS-based metabonomics technique combined with multivariate statistical analysis methods was used to acquire and analyze the metabolite changes of liver tissues in normal control, model, and LCS groups. The system’s stability and repeatability were assessed by monitoring the UPLC-Q-TOF/MS metabolic profiling of QCP samples. It showed that the total ion chromatograms (TICs) of QCP samples acquired in ESI^+^ and ESI^−^ modes were tightly clustered (Supplementary Figure S5). Moreover, PCA score plots of all analyzed samples in the LCS group, model group, normal group, and QCP samples showed that QCP samples were concentrated in the middle of all samples and with good aggregation degrees (Supplementary Figure S6), illustrating that the analytical system was stable and reproductive.

#### 3.2.1 Multivariate Data Analysis

**FIGURE 7:**
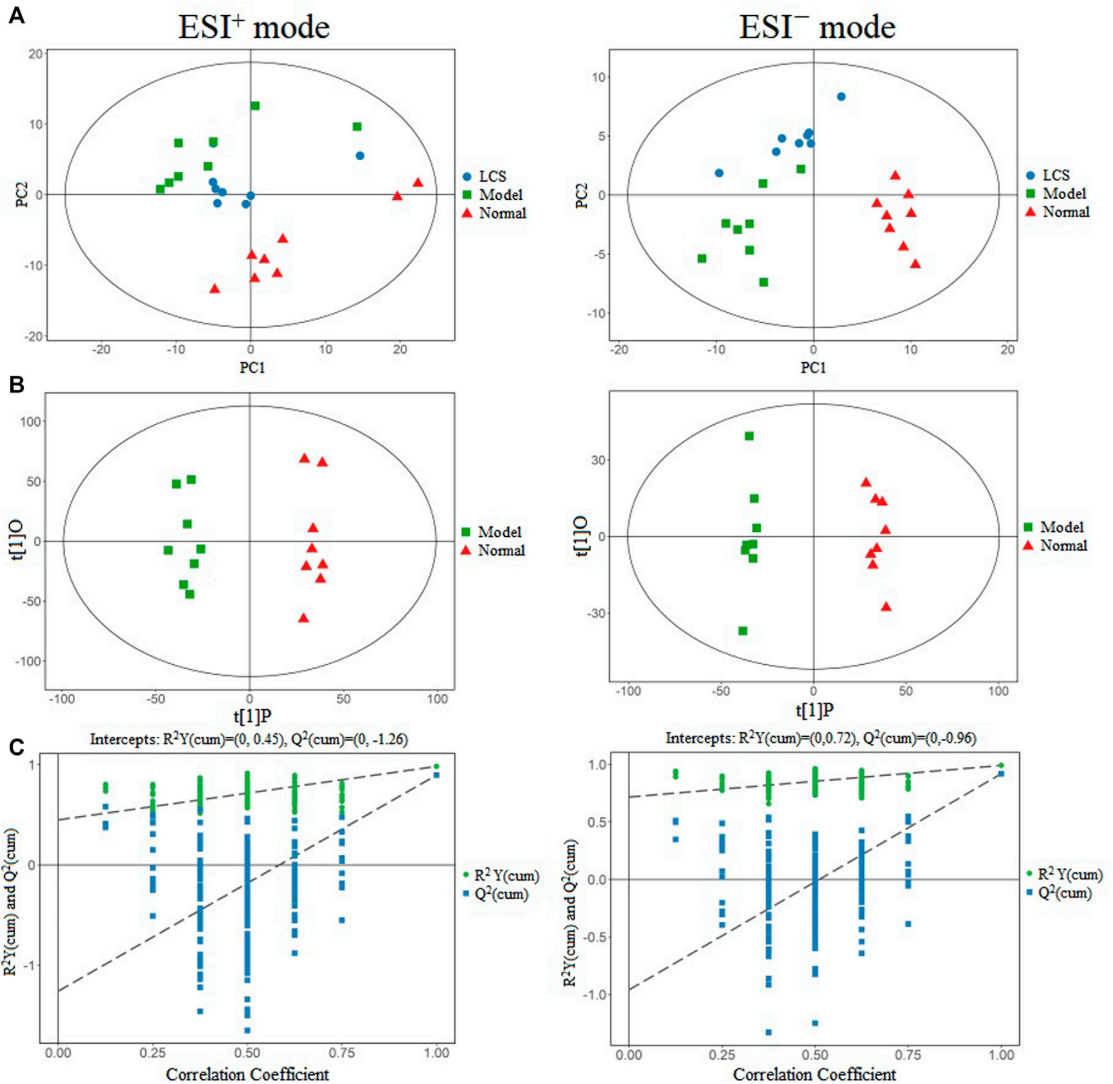
Multivariate data analysis of metabonomics for liver injury treatment of LCS. Note: **(A)** PCA score plots in positive ion mode and negative ion mode for the LCS, normal and model groups. **(B)** OPLS-DA score plots in positive ion mode and negative ion mode for the normal and model groups. **(C)** The 200-permutation test of OPLS-DA model for the normal and model groups.

The score plots of PCA displayed pattern distinction for the LCS group, model group, and normal control group in ESI^+^ as well as in ESI^−^ modes in their UPLC-Q-TOF/MS metabolic profiles (Figure 7A). Samples in the same group almost clustered together and were within the 95% *Hotelling*’s T-squared ellipse, confirming that the variables observed in samples were biologically relevant. The samples in the model group could be significantly discriminated against those of the normal control group, indicating that CCl_4_ could induce metabolic disorder in the liver. Furthermore, the PCA score plots also showed that the LCS group displayed a returning trend to the normal control group from that of the model group, showcasing the ability of LCS to reverse CCl_4_-induced metabolism disorder.

The datasets of samples from the normal control and model groups were extracted respectively as a matrix for OPLS-DA analysis to screen for potential differential metabolites associated with the liver injury induced by CCl_4_. The score plots for OPLS-DA presented obvious discrimination between the normal control group and the model group in the ESI^+^ data set as well as in the ESI^−^ data set (Figure 7B), indicating CCl_4_ could induce metabolic variations. The goodness-of-fit and predictability of OPLS-DA analysis models were evaluated by calculating R^2^Y and Q^2^, respectively. The result of the 200-permutation test of the OPLS-DA model indicated that the OPLS-DA model was reliable and not the result of statistical over-fitting (Figure 7C).

#### 3.2.2 Potential Biomarker Identification

The potential metabolites that significantly contributed to the clustering and discrimination were selected based on their variable importance projection (VIP) value and *p*-value. Metabolites with VIP >1 combined with *p* < 0.05 were selected as potential differential metabolites. Following the criteria aforementioned, 32 differential metabolites were identified (Table 1).

**TABLE 1:** Differential metabolites between the normal and model groups.

No.	*R*_*t*_ (s)	*m*/*z*	VIP	*p*-value	Fold change	Metabolites	Formula	ESI Mode	KEGG ID
1	43.67	279.23	1.11	0.02	1.61	Linoleic acid	C_18_H_32_O_2_	neg	C01595
2	44.23	277.22	1.25	0.01	2.18	*γ*-Linolenic acid	C_18_H_30_O_2_	neg	C06426
3	44.38	283.26	1.21	0.01	1.49	Stearic acid	C_18_H_36_O_2_	neg	C01530
4	44.44	253.22	1.55	0.01	0.33	Palmitoleic acid	C_16_H_30_O_2_	neg	C08362
5	104.66	241.08	1.72	0.00	3.35	Thymidine	C_10_H_14_N_2_O_5_	neg	C00214
6	160.84	113.03	1.75	0.00	1.42	Uracil	C_4_H_4_N_2_O_2_	pos	C00106
7	162.67	191.02	1.32	0.00	0.55	Isocitrate	C_6_H_8_O_7_	neg	C00311
8	168.25	114.06	1.15	0.04	1.19	Creatinine	C_4_H_7_N_3_O	pos	C00791
9	168.96	136.06	1.05	0.03	0.70	Adenine	C_5_H_5_N_5_	pos	C00147
10	173.56	266.08	1.62	0.00	0.21	Adenosine	C_10_H_13_N_5_O_4_	neg	C00212
11	203.08	151.03	1.40	0.00	1.65	Xanthine	C_5_H_4_N_4_O_2_	neg	C00385
12	203.71	228.10	1.34	0.01	1.24	Deoxycytidine	C_9_H_13_N_3_O_4_	pos	C00881
13	235.33	242.08	1.67	0.00	2.85	Cytidine	C_9_H_13_N_3_O_5_	neg	C00475
14	235.98	248.17	1.38	0.01	1.54	Taurocyamine phosphate	C_3_H_10_N_3_O_6_PS	pos	C03149
15	263.47	104.11	1.21	0.02	1.22	Choline	C_5_H_14_NO	pos	C00114
16	269.31	118.09	1.16	0.02	1.38	Betaine	C_5_H_11_NO_2_	pos	C00719
17	289.21	124.01	1.52	0.00	0.57	Taurine	C_2_H_7_NO_3_S	neg	C00245
18	290.85	128.04	1.45	0.00	1.45	5-Oxo-*L*-proline	C_5_H_7_NO_3_	neg	C01879
19	295.11	150.06	1.38	0.01	0.76	Methionine	C_5_H_11_NO_2_S	pos	C00073
20	331.68	165.04	1.22	0.00	0.60	7-Methylxanthine	C_6_H_6_N_4_O_2_	neg	C16353
21	336.29	247.06	1.84	0.00	0.35	Phosphatidyl glycerol	C_8_H_13_O_10_PR_2_	pos	C00344
22	341.57	136.05	1.45	0.01	1.61	Homocysteine	C_4_H_9_NO_2_S	pos	C05330
23	378.65	258.11	1.57	0.00	0.48	Glycerophosphocholine	C_8_H_21_NO_6_P+	pos	C00670
24	387.05	146.05	1.68	0.00	1.52	*L*-Glutamate	C_5_H_9_NO_4_	neg	C00025
25	390.72	383.11	1.49	0.00	1.36	*S*-Adenosyl-*L*-homocysteine	C_14_H_20_N_6_O_5_S	neg	C00021
26	395.83	306.08	1.18	0.01	0.50	Glutathione	C_10_H_17_N_3_O_6_S	neg	C00051
27	424.48	662.10	1.20	0.00	0.60	Nicotinamide adenine dinucleotide	C_21_H_28_N_7_O_14_P_2_	neg	C00003
28	433.00	489.12	1.28	0.01	0.60	CDP-choline	C_14_H_26_N_4_O_11_P_2_	pos	C00307
29	449.44	140.01	1.66	0.00	2.68	Phosphoethanolamine	C2H8NO4P	neg	C00346
30	459.80	261.04	1.75	0.00	0.51	*D*-Glucose-6-phosphate	C_6_H_13_O_9_P	pos	C00092
31	473.41	221.06	1.56	0.00	2.43	Cystathionine	C_7_H_14_N_2_O_4_S	neg	C02291
32	507.03	399.14	1.19	0.02	0.67	*S*-Adenosylmethionine	C_15_H_22_N_6_O_5_S	pos	C00019

Note: VIP values were obtained from the OPLS-DA model. Fold change (FC) was calculated by peak intensity. *p*-values were calculated by one-way ANOVA.

**FIGURE 8:**
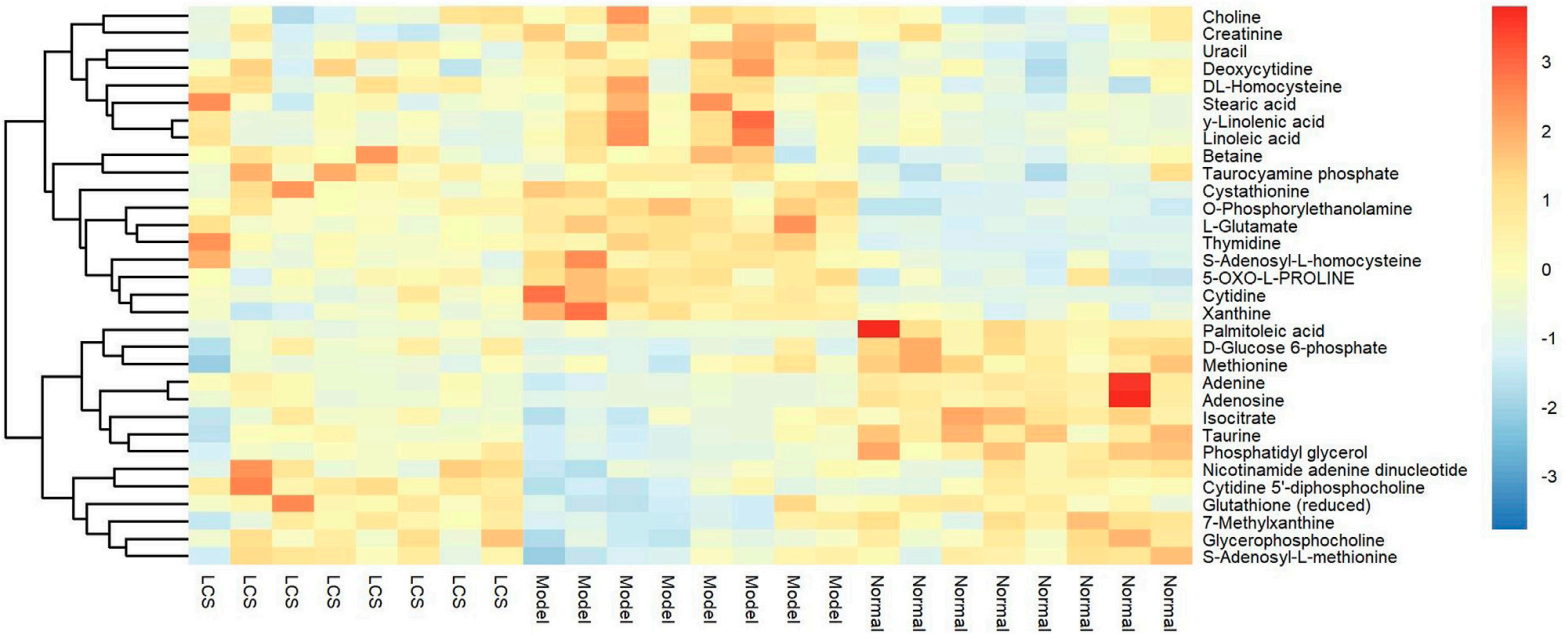
Heat-map of the relative intensity of hepatic differential metabolites treated by LCS. Note: Red and blue indicate increased and decreased levels, respectively.

**FIGURE 9:**
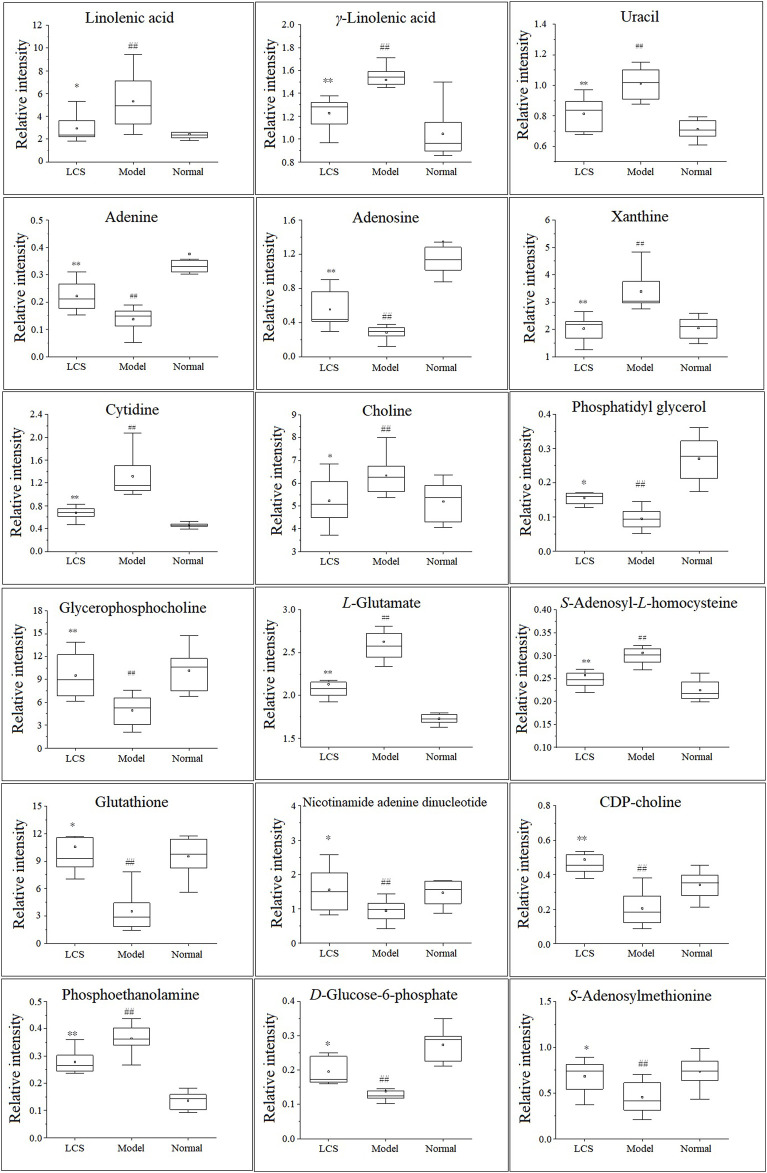
Eighteen hepatic biomarkers reversed by LCS treatment. Note: ^##^ *p* < 0.01 compared with the normal control group; ^**^ *p* < 0.01, ^*^ *p* < 0.05 compared with the model group.

In order to study the effect of LCS on disturbed metabolites induced by CCl_4_, a heat-map was drawn according to the relative intensity of 32 metabolites in LCS, normal, and model groups (Figure 8). The results of the heat-map analysis indicated that metabolites in the LCS group were similar to those in the normal control group. Meanwhile, the relative intensities of these metabolites in different groups were statistically analyzed by one-way ANOVA. As a result, 18 potential biomarkers were notably reversed by LCS treatment (Figure 9).

#### 3.2.3 Metabolic Pathway Analysis of Differential Metabolites

**FIGURE 10:**
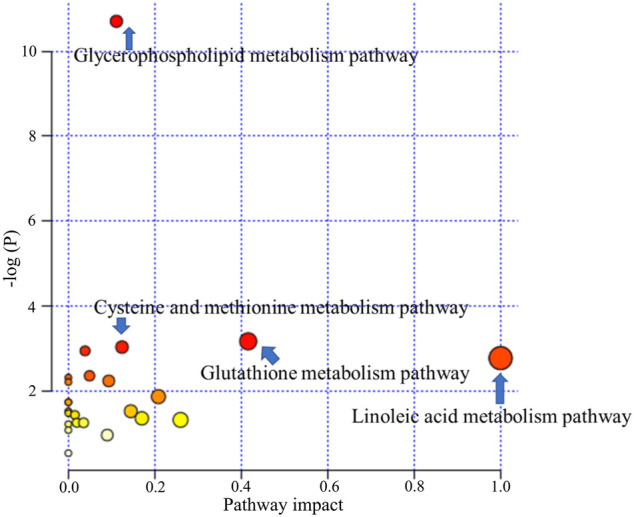
Metabolism pathways related to liver injury and LCS treatment.

To investigate the metabolism pathways influenced by LCS treatment, the significant differential metabolites were analyzed using Metaboanalyst 4.0. Ten corresponding pathways were highly related to LCS treatment (Figure 10). Among these metabolic pathways, linoleic acid metabolism, glutathione metabolism, cysteine and methionine metabolism, and glycerophospholipid metabolism pathways were filtered out as significant pathways since these pathways had impact values >0.10 and *p* < 0.05 (Hoffmann et al., 2014). The detailed information of these 4 significant metabolism pathways is shown in Supplementary Table S5.

Linoleic acid metabolism pathway: As a kind of free polyunsaturated fatty acid (PUFA), linoleic acid, and γ-linolenic acid, the precursors of many polyunsaturated fatty acids, are closely related to human health. The increases of these PUFA could reflect the outbreak of inflammatory response and oxidative stress during liver injury. After treatment with LCS, the increased hepatic linoleic acid and γ-linolenic acid levels could be significantly down-regulated, suggesting that LCS could modulate inflammatory responses and oxidative stress during liver injury by regulating the linoleic acid metabolism pathway.

Glutathione metabolism pathway: Glutathione plays an important role in eliminating free radicals and in defending oxidative stress and therefore decreasing liver injury (Gabele et al., 2009). In this study, we detected many metabolites involved in the glutathione metabolism pathway, such as glutathione and *L*-glutamate. LCS could increase the glutathione content in the CCl_4_-induced hepatic injury rats, suggesting that LCS had the ability to alleviate the disordered glutathione homeostasis.

Cysteine and methionine metabolism pathway: It is recognized that the liver plays an important role in cysteine and methionine metabolism, and marked impairment of this pathway is usually observed in patients with liver injuries. As one of the most important biochemical reactions, the cysteine and methionine metabolism generates approximately 70% methyl donors. In the present study, we detected that two metabolites, *S*-adenosyl-*L*-homocysteine (SAMe) and *S*-adenosylmethionine (SAH), involved in the methionine cycle reaction, were significantly decreased upon CCl_4_ inducement. SAMe has the capacity to increase glutathione levels, regulate the hepatocyte apoptotic response and membrane fluidity, and decrease the production of inflammatory factors, which has also been found to protect the liver against various injuries (Mato and Lu, 2007). SAH is a potent inhibitor of SAMe-dependent methylation reactions. It is toxic to immature lymphocytes and can lead to immune suppression. In our study, LCS had the ability to regulate the disordered cysteine and methionine pathway in the liver by reversing the levels of hepatic SAMe and SAH.

Glycerophospholipid metabolism pathway: Glycerophospholipids, storage deposits for lipid mediators, function as integral membrane proteins, transporters, receptors, and ion channels. As important intermediates involved in the synthesis of the characteristic bilayer structure of cells and the maintenance of membrane integrity, their perturbation reflects the disorders of lipid metabolism under severe oxidative stress (Ekroos, 2012). In our study, compared with the normal control group, the levels of glycerophospholipid metabolites (including phosphatidylglycerol, phosphoethanolamine, CDP-choline, and glycerophosphocholine) decreased in the model group, revealing that CCl_4_ could cause liver damage by altering the structural integrity and permeability of the plasma membrane in liver tissue. After treatment with LCS, the levels of glycerophospholipid metabolites were significantly reversed, suggesting that LCS could protect the cell membrane against oxidative stress damage.

### 3.3 Experimental Validation

Based on the results of network pharmacology and metabonomics, we focused on their shared main pathways to validate the potential mechanisms of LCS against liver injuries. Linoleic acid metabolism and glutathione metabolism pathways were two key common pathways that emerged in both network pharmacology and metabonomics analysis. It was reported that the linoleic acid metabolism pathway was relevant to inflammatory processes, and the increase in linoleic acid may reflect not only the outbreak of inflammatory response but also the oxidative stress during liver injury (Tufvesson et al., 2015). Liver injury could cause a serious inflammatory reaction in the liver, so the regulation of inflammation is important. As for the glutathione metabolism pathway, when oxidative stress is generated in the human body, the reduced glutathione would decline, lipid peroxidation would be induced, and corresponding free radicals would be generated (Maddrey, 2005). Glutathione may play a vital role in eliminating free radicals and in defending oxidative stress and thereby decreasing liver injury (Gabele et al., 2009). In the present study, experimental validation was conducted to investigate whether LCS could inhibit liver injuries by inhibiting inflammatory reactions, improving the antioxidant defense system, and suppressing lipid peroxidation by assessing the levels of inflammatory cytokines TNF-α, IL-6, IL-1β, and NO; the levels of antioxidant enzymes SOD, CAT, and GSH-Px; and the level of lipid peroxide MDA, respectively.

#### 3.3.1 Validation of Inhibiting Inflammatory Cytokines of Le-Cao-Shi

**FIGURE 11:**
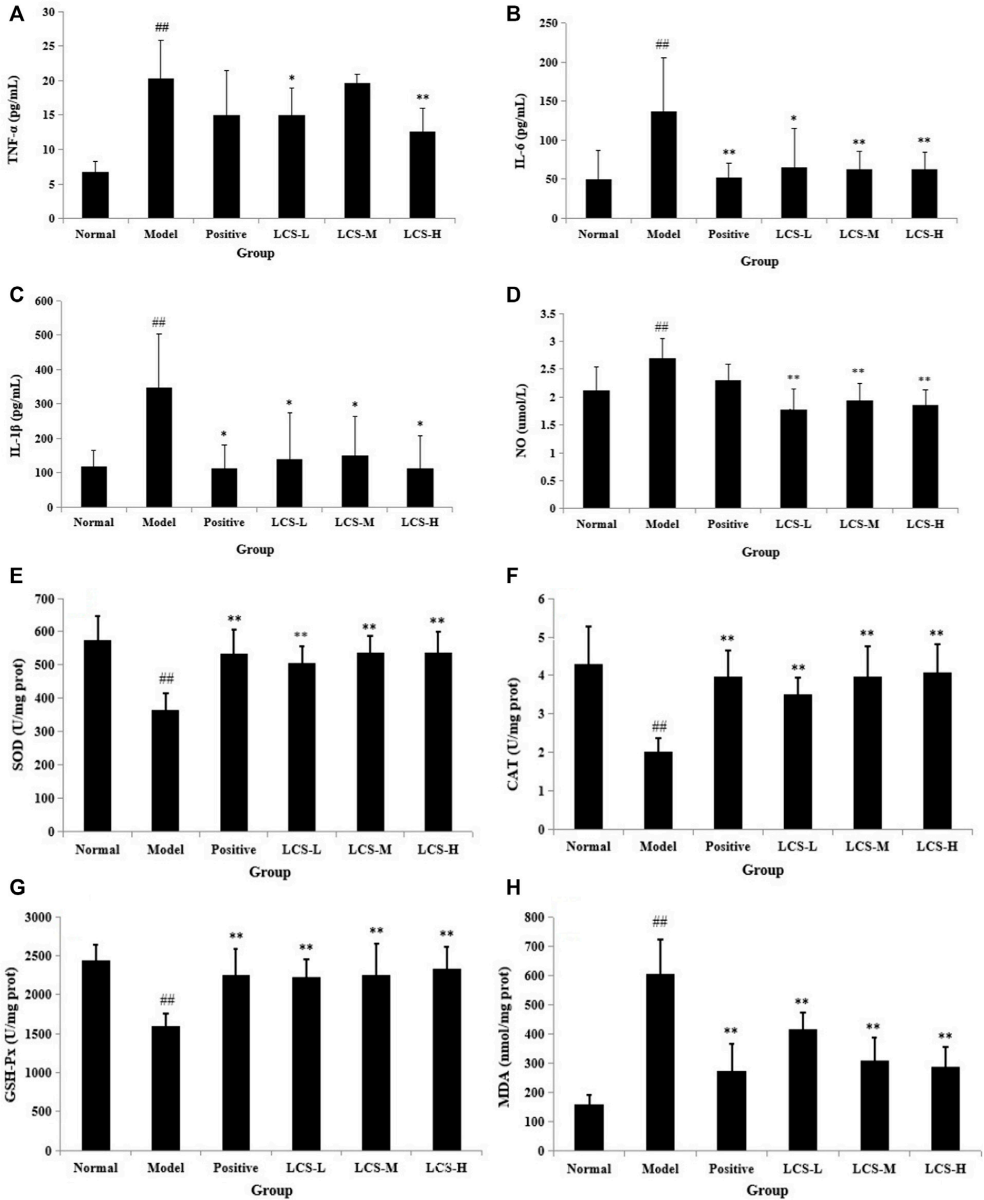
Levels of biochemical indexes in rat serum treated by LCS. **(A)** TNF-α, **(B)** IL-6, **(C)** IL-1β, **(D)** NO, **(E)** SOD, **(F)** CAT, **(G)** GSH-Px, and **(H)** MDA in rat serum treated by LCS. Note: The results are expressed as mean ±SD. ^##^ Statistically significant differences *p* < 0.01, compared with the normal control group; ^**^ statistically significant differences *p* < 0.01, compared with the model group; ^*^ statistically significant differences *p* < 0.05, compared with the model group.

Free radicals generated from CCl_4_ stimulated hepatocytes could secrete signals to activate the innate immune system and Kupffer cells, and then exacerbate liver inflammation by generating a variety of inflammatory cytokines, such as TNF-α, IL-6, and IL-1β (Lin et al., 2012; Wang et al., 2019). TNF-α stimulates the release of cytokines from macrophages and induces oxidative metabolism and NO production (Gao et al., 2014). NO is a highly reactive oxidant and its excessive accumulation could cause damage to the liver. Acute hepatotoxicity could induce inflammation mediated by cytokines. In order to determine the effect of LCS against CCl_4_-induced inflammation, the levels of cytokines (TNF-α, IL-6, IL-1β, and NO) in rat serum were measured. Results showed that the levels of these cytokines were significantly increased in the model group compared with the normal control group (*p* < 0.01, Figures 11A–D). In comparison with the model group, LCS exhibited significant inhibition effects on the secretion of these cytokines, especially IL-6 and IL-1β, with the levels about half of those of the model groups (*p* < 0.01, *p* < 0.05) and near to those of normal and positive groups (Figures 11B–C), indicating that LCS could inhibit the production of inflammatory cytokines in rat liver injury induced by CCl_4_.

#### 3.3.2 Validation of Activating Antioxidant Enzymes of Le-Cao-Shi

Oxidative stress is the main response of acute liver injury induced by CCl_4_, which is associated with the severity of lipid peroxidation and the activities of the antioxidant defense system (Karakus et al., 2011; Ma et al., 2015). A defense system of antioxidant enzymes plays an important role in preventing liver damage induced by CCl_4_ (Ma et al., 2015). Lipid peroxides and reactive radicals can easily inactivate antioxidant enzymes under toxic conditions. Therefore, changes in the hepatic levels of these antioxidant enzyme activities are closely related to the ability of the liver to cope with oxidative stress during CCl_4_ inducement. SOD, CAT, and GSH-Px are major enzymes in the antioxidant system (Zhong et al., 2009; Gao et al., 2011). Among them, SOD can convert superoxide anions into hydrogen peroxide, and CAT can catalyze the breakdown of hydrogen peroxide into oxygen and H_2_O, while GSH-Px can catalyze the reduction of hydrogen peroxide into non-toxic hydroxyl molecules (Hsu et al., 2008). In addition, as an index of the status of oxidative stress and lipid peroxidation, the end-product of lipid peroxidation, MDA, was broadly adopted to assess the level of lipid peroxidation (Abuja and Albertini, 2001). Herein, we assessed the activation of the three antioxidant enzymes (SOD, CAT, and GSH-Px) and the level of lipid peroxide MDA.

As shown in Figures 11E–G, the activities of hepatic SOD, CAT, and GSH-Px in the model group were significantly lower than those in the normal control group (*p* < 0.01). In comparison with the model group, the activities of SOD, CAT, and GSH-Px in LCS groups were significantly increased (*p* < 0.01) and were near to normal and positive groups. Specifically, all of the three dosages of LCS groups showed equal or superior abilities to increase the activity of GSH-Px in the positive group. Moreover, the level of MDA significantly increased in the model group compared to that in the normal control group (*p* < 0.01, Figure 11H). In comparison with the model group, the levels of MDA in the LCS groups were markedly reduced (*p* < 0.01), especially in the LCS-M and LCS-H groups, showing stronger reduction effects and close to the positive group. These results suggested that LCS could maintain antioxidant enzyme activities and ameliorate lipid peroxidation levels, which could improve the antioxidant defense system to attenuate liver injury induced by CCl_4_.

## Conclusion

In this study, an integrative approach of network pharmacology and metabonomics was performed to investigate the underlying mechanisms of LCS in treating liver injury. By network pharmacology, 57 candidate compounds and their 87 corresponding liver injury-related targets were selected and predicted. More than 15 pathways connected to these potential targets were found to be relevant to cancer, xenobiotic metabolism by cytochrome P450, bile metabolism, inflammation, and antioxidation modules. By CI calculation, 8 active compounds with accumulative CI values of 90% were identified displaying the greatest contribution to the anti-liver injury effects of LCS. By metabonomics based on UPLC-Q-TOF/MS, 32 differential metabolites were identified by comparing the normal and model groups. Among these metabolites, eighteen potential biomarkers could be regulated by LCS, which are closely related to linoleic acid metabolism, glutathione metabolism, cysteine and methionine metabolism, and glycerophospholipid metabolism pathways. Linoleic acid metabolism and glutathione metabolism pathways were found to be two key common pathways in both network pharmacology and metabonomics. These two pathways are tightly relevant to inflammatory, lipid peroxidation, and antioxidant processes. By experimental validation, TNF-α, IL-6, and IL-1β—the inflammatory parameters—could be significantly reduced. MDA, the end-product of lipid peroxidation, could be decreased, while SOD, CAT, and GSH-Px—the hepatic antioxidant enzymes—were found to be enhanced by LCS, indicating that LCS could inhibit liver injuries through anti-inflammatory, suppressing lipid peroxidation, and improving the antioxidant defense system. It could be concluded that the action mechanisms of LCS in treating liver injury were mainly attributed to the activation of the linoleic acid metabolism pathway and glutathione metabolism pathway to exert anti-inflammatory, lipid peroxidation suppression, and antioxidant defense system enhancement effects. Our study could help decipher the pharmacology mechanisms of LCS against liver injury, which would be beneficial for its further development in the treatment of liver injury diseases.

## Data Availability

The original contributions presented in the study are included in the article/Supplementary Material, further inquiries can be directed to the corresponding author.
