# Supplementary material for: Deciphering the Underlying Mechanisms of Formula Le-Cao-Shi Against Liver Injuries by Integrating Network Pharmacology, Metabonomics, and Experimental Validation
Source: Front Pharmacol. 2022 Apr 25;13:884480. doi: 10.3389/fphar.2022.884480 (PMC9081656; doi:10.3389/fphar.2022.884480)
Supplement: Supplementary file 1 [file DataSheet1.doc]

Supplementary Material

**Deciphering the underlying mechanisms of formula Le-Cao-Shi against liver injuries by integrating network pharmacology, metabonomics, and** **experimental validation**

**Qing Zhao^1,2^****^,†^, Xia Ren^1,2,†^, Shu-Yue Song^1,2^, Ri-Lei Yu^1,2^, Xin Li^1,2^, Peng Zhang^1,2^, Chang-Lun Shao^1,2*^, Chang-Yun Wang^1,2*^**

^1^*Key Laboratory of Marine Drugs, the Ministry of Education of China, School of Medicine and Pharmacy, Ocean University of China, Qingdao 266003, P. R. China*

^2^*Laboratory for Marine Drugs and Bioproducts, Qingdao National Laboratory for Marine Science and Technology, Qingdao 266237,* *P. R. China*

**†** These authors contributed equally to this work

*** Correspondence:**

Chang-Yun Wang

changyun@ouc.edu.cn

Chang-Lun Shao

shaochanglun@ouc.edu.cn

Corresponding authors

**Legends of Supplementary Figures**

Figure S1 Flowchart of network pharmacology analysis for LCS

**Figure S2** Structures of 126 compounds in *Acanthus ilicifolius*

**Figure S3** Structures of 50 compounds in *Phyllodium pulchellum*

**Figure S4** Structures of 43 compounds in *Cudrania cochinchinensis*

**Figure S5** Total ion chromatograms (TIC) of QCP samples acquired in ESI^+^ (A) and ESI^−^ (B) mode

**Figure S6** PCA score plots of all analyzed samples in the LCS group, model group, normal group and QCP samples

**Legends of Supplementary Tables**

**Table S1** The ADME parameters of 57 candidate compounds in LCS

**Table S2** The information of 87 potential targets

**Table S3** The number of targets and reference, network based efficacy (NE), and contribution index (CI) values of active compounds in LCS

**Table S4** The molecular docking results of 40 representative targets

**Table S5** Detailed information of four significant metabolism pathways


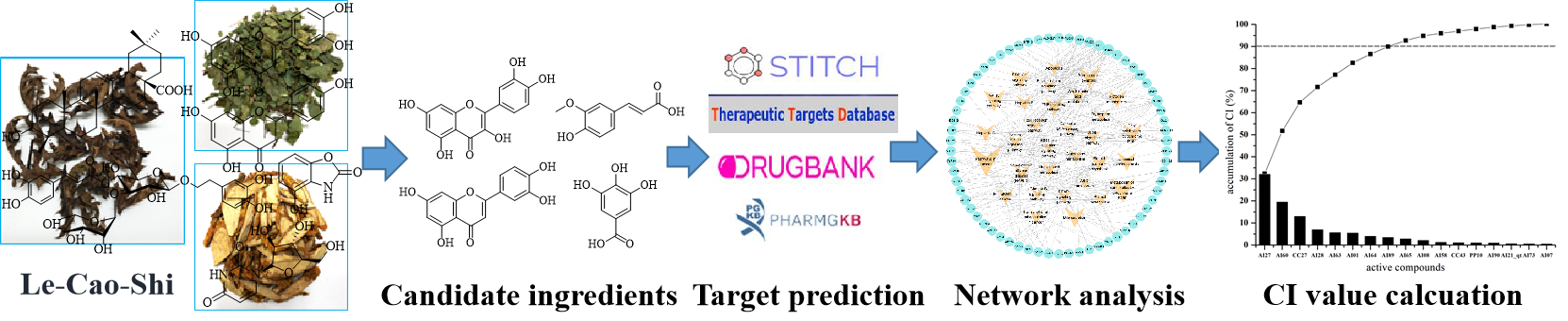


Figure S1 Flowchart of network pharmacology analysis for LCS

**Figure S2** Structures of 126 compounds in *Acanthus ilicifolius*

**Figure S3** Structures of 50 compounds in *Phyllodium pulchellum*

**Figure S4** Structures of 43 compounds in *Cudrania cochinchinensis*


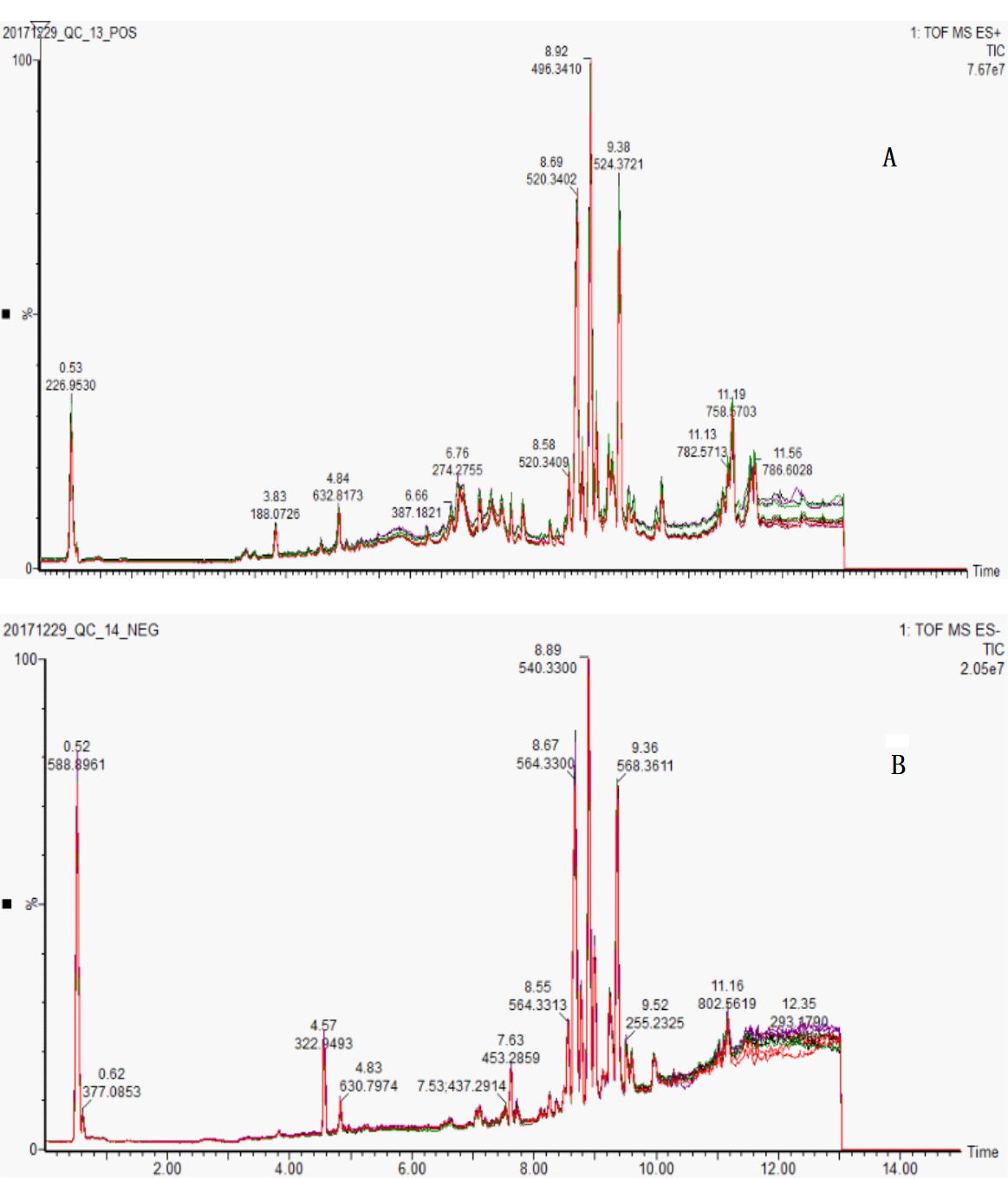


**Figure S5** Total ion chromatograms (TIC) of QCP samples acquired in ESI^+^ (A) and ESI^−^ (B) mode


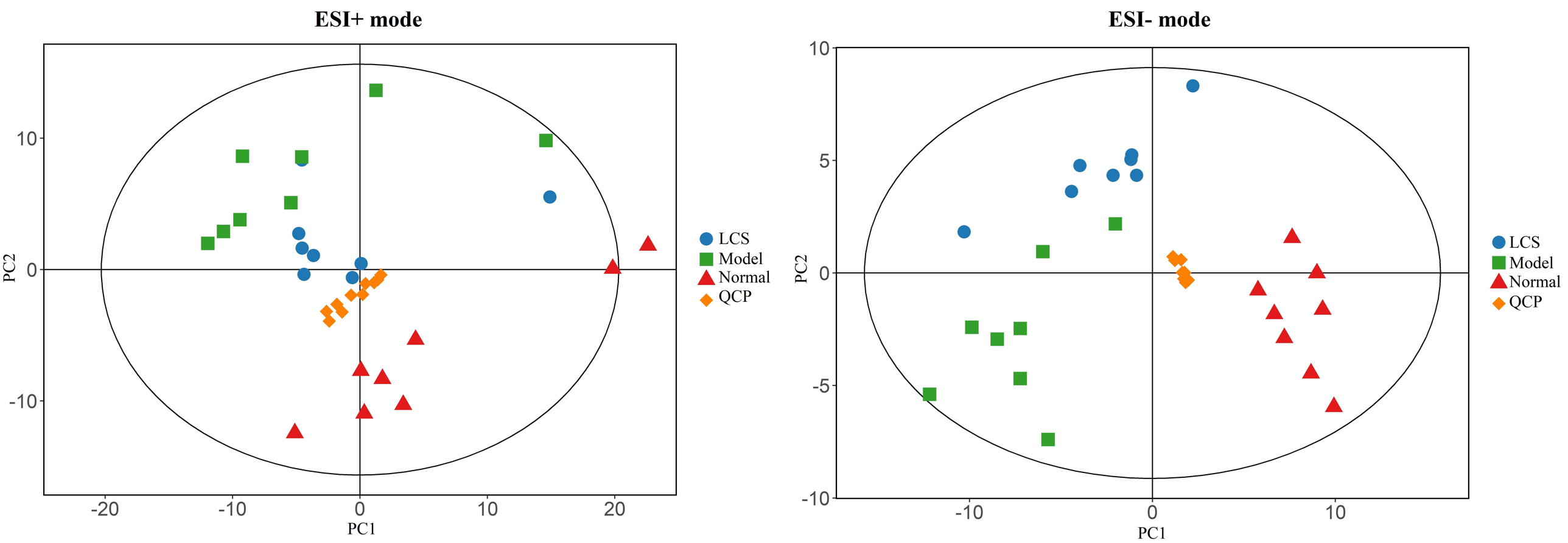


**Figure S6** PCA score plots of all analyzed samples in the LCS group, model group, normal group and QCP samples

**Table S1** The ADME parameters of 57 candidate compounds in LCS

| No. | Compound | CAS | OB(%) | Caco-2 | DL |
| --- | --- | --- | --- | --- | --- |
| AI01 | oleanolic acid | 508-02-1 | 29.02 | 0.59 | 0.76 |
| AI03 | *α*-amyrin | 638-95-9 | 39.51 | 1.42 | 0.76 |
| AI05 | campesterol | 474-62-4 | 37.58 | 1.34 | 0.71 |
| AI06 | cholesterol | 57-88-5 | 37.87 | 1.43 | 0.68 |
| AI07 | stigmasterol | 83-48-7 | 43.83 | 1.44 | 0.76 |
| AI08 | *β*-sitosterol | 83-46-5 | 36.91 | 1.32 | 0.75 |
| AI10 | 3*β*-hydroxyl-stigmasta-5,22-diene-7-one | N/A | 43.04 | 1.35 | 0.82 |
| AI12 | cycloartenol | 469-38-5 | 38.69 | 1.53 | 0.78 |
| AI16 | stigmasta-4,22-diene-3,6-dione | 50868-51-4 | 39.32 | 0.72 | 0.79 |
| AI21_qt | stigmasterol glucoside_qt | 19716-26-8 | 43.83 | 1.31 | 0.76 |
| AI22_qt | *β*-sitosterol-3-*O*-*β*-D glucopyranoside_qt | 474-58-8 | 36.91 | 1.32 | 0.75 |
| AI27/PP02/CC28 | quercetin | 117-39-5 | 46.43 | 0.05 | 0.28 |
| AI28 | luteolin | 491-70-3 | 36.16 | 0.19 | 0.25 |
| AI36 | 2-benzoxazolinone | 59-49-4 | 63.01 | 0.72 | 0.05 |
| AI37 | 4-hydroxy-2-benzoxazolone | 140623-61-6 | N/A | N/A | N/A |
| AI41 | blepharigenin | 23520-34-5 | N/A | N/A | N/A |
| AI42 | (*2R*)-2-*O*-β-D-glucopyranosyl-*2H*-1,4-benzoxazin-3(*4H*)-one | 27625-86-1 /23520-34-5 | N/A | N/A | N/A |
| AI45 | (2*R*)-2-*O*-*β*-*D*-glucopyranosyl-5-hydroxy-2*H*-1,4-benzoxazin-3(4*H*)-one | N/A | N/A | N/A | N/A |
| AI47 | 7-Cl-(2R)-2-O-β-D-glucopyranosyl-2H-1,4-benzoxazin-3(4H)-one | N/A | N/A | N/A | N/A |
| AI49 | uracil | 66-22-8 | 42.53 | 0.05 | 0.02 |
| AI53 | adenosine | 58-61-7 | 4.28 | ‒1.73 | 0.21 |
| AI54 | trigonellin | 535-83-1 | 60.07 | 0.58 | 0.03 |
| AI55 | *L*-proline | 147-85-3 | 77.57 | 0.22 | 0.01 |
| AI56 | 1H-indole-3-carboxylic acid | 771-50-6 | 33.86 | 0.82 | 0.05 |
| AI58 | 1*H*-indole-3-acetic acid | 87-51-4 | 46.15 | 0.70 | 0.06 |
| AI60 | ferulic acid | 1135-24-6 | 39.56 | 0.47 | 0.06 |
| AI61/PP40 | *p*-coumaric acid | 501-98-4 | 43.29 | 0.46 | 0.04 |
| AI62/PP34 | 4-Hydroxybenzoic acid, PHB | 99-96-7/117-39-5 | 30.15 | 0.39 | 0.03 |
| AI63 | gallic acid | 149-91-7 | 31.69 | ‒0.09 | 0.04 |
| AI64 | vanillic acid | 557-61-9 | 35.47 | 0.43 | 0.04 |
| AI65 | syringic acid | 530-57-4 | 47.78 | 0.50 | 0.06 |
| AI71 | 2,6-dimethoxy-p-hydroquinone 1-*O*-*β*-D-glucopyranoside | N/A | N/A | N/A | N/A |
| AI73/PP43 | loliolide | 11028-27-6 | 44.66 | 0.54 | 0.08 |
| AI89 | acteoside | 61276-17-3 | 2.94 | ‒1.89 | 0.62 |
| AI90 | isoacteoside | 61303-13-7 | 3.05 | ‒1.56 | 0.61 |
| AI93 | ampneosides II | 95587-86-3 | 3.30 | ‒2.39 | 0.59 |
| AI107 | aurantiamide acetate | 56121-42-7 | 58.02 | 0.28 | 0.52 |
| AI109 | hexane | 110-54-3 | 59.94 | 1.81 | 0.01 |
| AI110 | squalene | 111-02-4 | 33.55 | 2.08 | 0.42 |
| PP09 | (+)-catechin | 638-95-9 | 54.83 | ‒0.03 | 0.24 |
| PP10 | (-)-epicatechin | 474-62-4 | 28.93 | ‒0.03 | 0.24 |
| PP11 | (-)-gallocatechin | 57-88-5 | 18.57 | ‒0.34 | 0.27 |
| PP12 | (-)-epigallocatechin | 83-48-7 | 24.18 | ‒0.22 | 0.27 |
| PP14 | gramine | 83-46-5 | 60.14 | 1.58 | 0.05 |
| PP18 | 5-methoxy-N,N-dimethyltryptamine | N/A | 63.78 | 1.66 | 0.09 |
| PP20 | tryptamine | 469-38-5 | 32.08 | 1.03 | 0.05 |
| PP25 | *L*-tryptophan | 50868-51-4 | 75.93 | 0.26 | 0.08 |
| PP26 | N,N-dimethyl-*L*-tryptophan | 19716-26-8 | 85.06 | 0.01 | 0.19 |
| PP32 | 1-methyl-2,3,4,9-tetrahydro-1H-pyrido[3,4-b]indole | 486-84-0 | 33.10 | 1.52 | 0.10 |
| PP36 | protocatechuic acid methyl ester | 491-70-3 | 38.19 | 0.48 | 0.04 |
| PP37 | protocatechuic acid ethyl ester | 59-49-4 | 35.77 | 0.59 | 0.05 |
| PP41 | caffeic acid ester | 23520-34-5 | 103.85 | 0.73 | 0.07 |
| CC27 | naringenin | 67604-48-2 | 43.29 | 0.46 | 0.04 |
| CC29 | 3,4',5,7-tetrahydroxyflavanone | 4049-38-1 | 71.79 | 0.17 | 0.24 |
| CC33 | wighteone | 51225-30-0 | 42.80 | 0.64 | 0.36 |
| CC38 | 3'-O-methylorobol | 36190-95-1 | 57.41 | 0.45 | 0.27 |
| CC43 | bergapten | 484-20-8 | 42.21 | 0.94 | 0.13 |

**Table S2** The information of 87 potential targets

| No. | Target Name | Gene name |
| --- | --- | --- |
| T1 | Multidrug resistance protein 1 | ABCB1 |
| T2 | ATP binding cassette subfamily B member11 | ABCB11 |
| T3 | Multidrug resistance protein 4 | ABCC4 |
| T4 | ATP-binding cassette subfamily G member 2 | ABCG2 |
| T5 | Low molecular weight phosphotyrosine protein phosphatase | ACP1 |
| T6 | Aldo-keto reductase family 1 member B10 | AKR1B10 |
| T7 | Aldo-keto reductase family 1 member C2 | AKR1C2 |
| T8 | Aldo-keto reductase family 1 member C3 | AKR1C3 |
| T9 | Aldo-keto reductase family 1 member C4 | AKR1C4 |
| T10 | RAC-alpha serine/threonine-protein kinase | AKT1 |
| T11 | Serum albumin | ALB |
| T12 | Arachidonate 12-lipoxygenase, 12S-type | ALOX12 |
| T13 | Arachidonate 15-lipoxygenase | ALOX15 |
| T14 | Arachidonate 5-lipoxygenase | ALOX5 |
| T15 | Amyloid beta A4 protein | APP |
| T16 | Carbonic anhydrase 1 | CA1 |
| T17 | Carbonic anhydrase 12 | CA12 |
| T18 | Carbonic anhydrase 13 | CA13 |
| T19 | Carbonic anhydrase 2 | CA2 |
| T20 | Carbonic anhydrase 3 | CA3 |
| T21 | Carbonic anhydrase 5A, mitochondria | CA5A |
| T22 | Carbonic anhydrase 5B, mitochondrial | CA5B |
| T23 | Carbonic anhydrase 6 | CA6 |
| T24 | Carbonic anhydrase 7 | CA7 |
| T25 | Caspase-3 | CASP3 |
| T26 | Carbonyl reductase [NADPH] 1 | CBR1 |
| T27 | M-phase inducer phosphatase 2 | CDC25B |
| T28 | Cyclin-dependent kinase 2 | CDK2 |
| T29 | Liver carboxylesterase 1 | CES1 |
| T30 | Cytochrome P450 1A1 | CYP1A1 |
| T31 | Cytochrome P450 1A2 | CYP1A2 |
| T32 | Cytochrome P450 1B1 | CYP1B1 |
| T33 | Cytochrome P450 2C9 | CYP2C9 |
| T34 | Cytochrome P450 2D6 | CYP2D6 |
| T35 | Cytochrome P450 3A4 | CYP3A4 |
| T36 | Epidermal growth factor receptor | EGFR |
| T37 | Prothrombin | F2 |
| T38 | Fatty acid-binding protein, epidermal | FABP5 |
| T39 | Fibroblast growth factor 1 | FGF1 |
| T40 | Vitamin D-binding protein | GC |
| T41 | mRNA of GLI2 | GLI2 |
| T42 | Lactoylglutathione lyase | GLO1 |
| T43 | G-protein coupled bile acid receptor 1 | GPBAR1 |
| T44 | Glutathione S-transferase A1 | GSTA1 |
| T45 | Glutathione S-transferase Mu 1 | GSTM1 |
| T46 | Glutathione S-transferase P | GSTP1 |
| T47 | Heparanase | HPSE |
| T48 | Corticosteroid 11-beta-dehydrogenase isozyme 1 | HSD11B1 |
| T49 | Corticosteroid 11-beta-dehydrogenase isozyme 2 | HSD11B2 |
| T50 | 5-hydroxytryptamine receptor 1D | HTR1D |
| T51 | Insulin-like growth factor-binding protein 3 | IGFBP3 |
| T52 | NF-κB essential modulator | IKBKG |
| T53 | Interleukin-1 beta | IL-1β |
| T54 | Interleukin-2 | IL-2 |
| T55 | Leukotriene B4 receptor 1 | LTB4R |
| T56 | Amine oxidase [flavin-containing] A | MAOA |
| T57 | Amine oxidase [flavin-containing] B | MAOB |
| T58 | Mitogen-activated protein kinase 14 | MAPK14 |
| T59 | Myoglobin | MB |
| T60 | Interstitial collagenase | MMP1 |
| T61 | 72 kDa type IV collagenase | MMP2 |
| T62 | Stromelysin-1 | MMP3 |
| T63 | Nuclear factor erythroid 2-related factor 2 | NFE2L2 |
| T64 | Nuclear factor NF-κB p105 subunit | NF-κB1 |
| T65 | NAD(P)H dehydrogenase [quinone] 1 | NQO1 |
| T66 | Bile acid receptor | NR1H4 |
| T67 | Ornithine decarboxylase | ODC1 |
| T68 | Peroxisome proliferator-activated receptor gamma | PPARγ |
| T69 | Protein kinase C beta type | PRKCβ |
| T70 | Tyrosine-protein phosphatase non-receptor type 1 | PTPN1 |
| T71 | Transcription factor p65 | RELA |
| T72 | Retinoic acid receptor RXR-alpha | RXR-α |
| T73 | Retinoic acid receptor RXR-beta | RXR-β |
| T74 | Retinoic acid receptor RXR-gamma | RXR-γ |
| T75 | L-selectin | SELL |
| T76 | Corticosteroid-binding globulin | SERPINA6 |
| T77 | Sodium/bile acid cotransporter | SLC10A1 |
| T78 | Ileal sodium/bile acid cotransporter | SLC10A2 |
| T79 | Sodium/glucose cotransporter 1 | SLC5A1 |
| T80 | Sodium/glucose cotransporter 2 | SLC5A2 |
| T81 | Solute carrier organic anion transporter family member 1B1 | SLCO1B1 |
| T82 | Tumor necrosis factor receptor superfamily member 1A | TNFRSF1A |
| T83 | DNA topoisomerase 2-alpha | TOP2A |
| T84 | Transthyretin | TTR |
| T85 | Tubulin beta-1 chain | TUBB1 |
| T86 | Vitamin D3 receptor | VDR |
| T87 | Vascular endothelial growth factor A | VEGFA |

**Table S3** The number of targets and reference, network based efficacy (NE), and contribution index (CI) values of active compounds in LCS

| No. | Comp. Name | No. targets | NE | No. Ref. | CI value (%) |
| --- | --- | --- | --- | --- | --- |
| AI27/PP02/CC28 | quercetin | 7 | 21 | 44 | 32.04 |
| AI60 | ferulic acid | 25 | 70 | 8 | 19.42 |
| CC27 | naringenin | 11 | 34 | 11 | 12.97 |
| AI28 | luteolin | 12 | 25 | 8 | 6.93 |
| AI63 | gallic acid | 9 | 10 | 16 | 5.55 |
| AI01 | oleanolic acid | 6 | 12 | 13 | 5.41 |
| AI64 | vanillic acid | 19 | 38 | 3 | 3.95 |
| AI89 | acteoside | 13 | 14 | 7 | 3.40 |
| AI65 | syringic acid | 14 | 20 | 4 | 2.77 |
| AI08 | *β*-sitosterol | 11 | 10 | 6 | 2.08 |
| AI58 | 1*H*-indole-3-acetic acid | 5 | 17 | 2 | 1.18 |
| CC43 | bergapten | 12 | 29 | 1 | 1.01 |
| PP10 | (-)-epicatechin | 12 | 27 | 1 | 0.94 |
| AI90 | isoacteoside | 11 | 13 | 2 | 0.90 |
| AI21_qt | stigmasterol glucoside_qt | 5 | 8 | 2 | 0.55 |
| AI73/PP43 | loliolide | 4 | 7 | 2 | 0.49 |
| AI07 | stigmasterol | 6 | 6 | 2 | 0.41 |
| PP41 | caffeic acid ester | 17 | 27 | 0 | 0 |
| AI93 | campneosides II | 9 | 13 | 0 | 0 |
| PP09 | (+)-catechin | 12 | 23 | 0 | 0 |
| PP12 | (-)-epigallocatechin | 4 | 13 | 0 | 0 |
| PP25 | *L*-tryptophan | 7 | 30 | 0 | 0 |
| AI62/PP34 | p-hydroxybenzoic acid | 14 | 22 | 0 | 0 |
| AI03 | *α*-amyrin | 5 | 7 | 0 | 0 |
| AI42 | (2R)-2-O-β-D-glucopyranosyl-2H-1,4-benzoxazin-3(4H)-one | 5 | 10 | 0 | 0 |
| PP26 | N,N-dimethyl-*L*-tryptophan | 7 | 23 | 0 | 0 |
| AI107 | aurantiamide acetate | 7 | 16 | 0 | 0 |
| PP37 | protocatechuic acid ethyl ester | 6 | 9 | 0 | 0 |
| PP36 | protocatechuic acid methyl ester | 9 | 11 | 0 | 0 |
| AI05 | campesterol | 8 | 5 | 0 | 0 |
| AI36 | 2-benzoxazolinone | 3 | 7 | 0 | 0 |
| AI06 | cholesterol | 10 | 10 | 0 | 0 |
| AI37 | 4-hydroxy-2-benzoxazolone | 1 | 0 | 0 | 0 |
| AI16 | stigmasta-4,22-diene-3,6-dione | 3 | 1 | 0 | 0 |
| AI10 | 3*β*-hydroxyl-stigmasta-5,22-diene-7-one | 5 | 4 | 0 | 0 |
| AI54 | trigonellin | 1 | 0 | 1 | 0 |
| AI109 | hexane | 1 | 0 | 0 | 0 |
| AI56 | 1H-indole-3-carboxylic acid | 6 | 12 | 0 | 0 |
| AI22_qt | *β*-sitosterol-3-O-β-D glucopyranoside | 9 | 10 | 0 | 0 |
| AI47 | 7-Cl-(2R)-2-O-β-D-glucopyranosyl-2H-1,4-benzoxazin-3(4H)-one | 4 | 6 | 0 | 0 |
| AI45 | (2*R*)-2-*O*-*β*-*D*-glucopyranosyl-5-hydroxy-2*H*-1,4-benzoxazin-3(4*H*)-one | 4 | 6 | 0 | 0 |
| PP14 | gramine | 5 | 16 | 0 | 0 |
| PP18 | 5-methoxy-N,N-dimethyltryptamine | 3 | 4 | 0 | 0 |
| PP20 | tryptamine | 3 | 20 | 0 | 0 |
| PP11 | (-)-gallocatechin | 4 | 13 | 0 | 0 |
| PP32 | 1-methyl-2,3,4,9-tetrahydro-1H-pyrido[3,4-b]indole | 7 | 20 | 0 | 0 |
| CC29 | 3,4',5,7-tetrahydroxyflavanone | 5 | 14 | 0 | 0 |
| CC33 | wighteone | 8 | 32 | 0 | 0 |

**Table S4** The molecular docking results of 40 representative targets

| No. | Target name | Uniprot ID | Gene name | Compound No. | Target PDB ID | The lowest free energy |
| --- | --- | --- | --- | --- | --- | --- |
| T2 | ATP binding cassette subfamily B member11 | O95342 | ABCB11 | AI08 | 6LR0 | ‒6.2938757 |
| T2 | ATP binding cassette subfamily B member11 | O95342 | ABCB11 | AI07 | 6LR0 | ‒5.8527937 |
| T2 | ATP binding cassette subfamily B member11 | O95342 | ABCB11 | AI10 | 6LR0 | ‒5.5508709 |
| T3 | Multidrug resistance protein 4 | O15439 | ABCC4 | AI21_qt | 4V1M | ‒6.3487029 |
| T3 | Multidrug resistance protein 4 | O15439 | ABCC4 | AI07 | 4V1M | ‒6.2300496 |
| T3 | Multidrug resistance protein 4 | O15439 | ABCC4 | AI08 | 4V1M | ‒6.1204815 |
| T3 | Multidrug resistance protein 4 | O15439 | ABCC4 | AI22_qt | 4V1M | ‒6.0118527 |
| T3 | Multidrug resistance protein 4 | O15439 | ABCC4 | AI06 | 4V1M | ‒5.7484498 |
| T3 | Multidrug resistance protein 4 | O15439 | ABCC4 | AI05 | 4V1M | ‒5.6959658 |
| T4 | ATP-binding cassette sub-family G member 2 | Q9UNQ0 | ABCG2 | CC38 | 6FFC | ‒8.6096315 |
| T4 | ATP-binding cassette sub-family G member 2 | Q9UNQ0 | ABCG2 | CC33 | 6FFC | ‒7.7787094 |
| T4 | ATP-binding cassette sub-family G member 2 | Q9UNQ0 | ABCG2 | CC27 | 6FFC | ‒6.8774548 |
| T4 | ATP-binding cassette sub-family G member 2 | Q9UNQ0 | ABCG2 | AI28 | 6FFC | ‒6.8236666 |
| T4 | ATP-binding cassette sub-family G member 2 | Q9UNQ0 | ABCG2 | CC43 | 6FFC | ‒6.7962236 |
| T4 | ATP-binding cassette sub-family G member 2 | Q9UNQ0 | ABCG2 | PP41 | 6FFC | ‒5.8450418 |
| T4 | ATP-binding cassette sub-family G member 2 | Q9UNQ0 | ABCG2 | AI65 | 6FFC | ‒5.6595664 |
| T4 | ATP-binding cassette sub-family G member 2 | Q9UNQ0 | ABCG2 | AI60 | 6FFC | ‒5.5984378 |
| T4 | ATP-binding cassette sub-family G member 2 | Q9UNQ0 | ABCG2 | AI61/PP40 | 6FFC | ‒5.0979552 |
| T4 | ATP-binding cassette sub-family G member 2 | Q9UNQ0 | ABCG2 | AI64 | 6FFC | ‒5.0200701 |
| T7 | Aldo-keto reductase family 1 member C2 | P52895 | AKR1C2 | PP26 | 4JTQ | ‒6.2777367 |
| T7 | Aldo-keto reductase family 1 member C2 | P52895 | AKR1C2 | AI65 | 4JTQ | ‒5.3659716 |
| T7 | Aldo-keto reductase family 1 member C2 | P52895 | AKR1C2 | AI64 | 4JTQ | ‒5.0231953 |
| T7 | Aldo-keto reductase family 1 member C2 | P52895 | AKR1C2 | AI56 | 4JTQ | ‒4.8139901 |
| T7 | Aldo-keto reductase family 1 member C2 | P52895 | AKR1C2 | AI63 | 4JTQ | ‒4.7529411 |
| T8 | Aldo-keto reductase family 1 member C3 | P42330 | AKR1C3 | AI73/PP43 | 5HNU | ‒5.9908929 |
| T8 | Aldo-keto reductase family 1 member C3 | P42330 | AKR1C3 | AI60 | 5HNU | ‒5.9700289 |
| T8 | Aldo-keto reductase family 1 member C3 | P42330 | AKR1C3 | AI65 | 5HNU | ‒5.7847419 |
| T8 | Aldo-keto reductase family 1 member C3 | P42330 | AKR1C3 | AI61/PP40 | 5HNU | ‒5.5279598 |
| T8 | Aldo-keto reductase family 1 member C3 | P42330 | AKR1C3 | AI64 | 5HNU | ‒5.4109874 |
| T8 | Aldo-keto reductase family 1 member C3 | P42330 | AKR1C3 | AI56 | 5HNU | ‒5.2161384 |
| T8 | Aldo-keto reductase family 1 member C3 | P42330 | AKR1C3 | AI62/PP34 | 5HNU | ‒4.9345999 |
| T9 | Aldo-keto reductase family 1 member C4 | P17516 | AKR1C4 | PP41 | 2FVL | ‒6.1323862 |
| T14 | Arachidonate 5-lipoxygense | P09915 | ALOX5 | AI28 | 3O8Y | ‒4.2756176 |
| T19 | Carbonic anhydrase 2 | P00918 | CA2 | PP10 | 3N76 | ‒7.2008858 |
| T19 | Carbonic anhydrase 2 | P00918 | CA2 | PP09 | 3N76 | ‒7.1902404 |
| T19 | Carbonic anhydrase 2 | P00918 | CA2 | AI28 | 3N76 | ‒6.9760976 |
| T19 | Carbonic anhydrase 2 | P00918 | CA2 | AI08 | 3N76 | ‒6.9137354 |
| T19 | Carbonic anhydrase 2 | P00918 | CA2 | PP37 | 3N76 | ‒5.7228947 |
| T19 | Carbonic anhydrase 2 | P00918 | CA2 | AI65 | 3N76 | ‒5.6829867 |
| T19 | Carbonic anhydrase 2 | P00918 | CA2 | AI60 | 3N76 | ‒5.3858819 |
| T19 | Carbonic anhydrase 2 | P00918 | CA2 | PP36 | 3N76 | ‒5.11058 |
| T19 | Carbonic anhydrase 2 | P00918 | CA2 | AI61 | 3N76 | ‒4.9941659 |
| T19 | Carbonic anhydrase 2 | P00918 | CA2 | AI63 | 3N76 | ‒4.9497142 |
| T19 | Carbonic anhydrase 2 | P00918 | CA2 | AI62/PP34 | 3N76 | ‒4.7399945 |
| T20 | Carbonic anhydrase 3 | P07451 | CA3 | AI27/PP02/CC28 | 1FLJ | ‒6.1669784 |
| T20 | Carbonic anhydrase 3 | P07451 | CA3 | CC43 | 1FLJ | ‒6.1672983 |
| T20 | Carbonic anhydrase 3 | P07451 | CA3 | CC29 | 1FLJ | ‒6.0691924 |
| T20 | Carbonic anhydrase 3 | P07451 | CA3 | CC27 | 1FLJ | ‒5.6706953 |
| T20 | Carbonic anhydrase 3 | P07451 | CA3 | PP11 | 1FLJ | ‒5.5024433 |
| T20 | Carbonic anhydrase 3 | P07451 | CA3 | PP12 | 1FLJ | ‒5.5005364 |
| T20 | Carbonic anhydrase 3 | P07451 | CA3 | PP09 | 1FLJ | ‒5.4314284 |
| T20 | Carbonic anhydrase 3 | P07451 | CA3 | PP10 | 1FLJ | ‒5.3332477 |
| T20 | Carbonic anhydrase 3 | P07451 | CA3 | AI65 | 1FLJ | ‒5.0844955 |
| T20 | Carbonic anhydrase 3 | P07451 | CA3 | AI64 | 1FLJ | ‒4.7997284 |
| T20 | Carbonic anhydrase 3 | P07451 | CA3 | AI61/PP40 | 1FLJ | ‒4.5955005 |
| T20 | Carbonic anhydrase 3 | P07451 | CA3 | AI63 | 1FLJ | ‒4.4711976 |
| T20 | Carbonic anhydrase 3 | P07451 | CA3 | AI36 | 1FLJ | ‒4.1855378 |
| T20 | Carbonic anhydrase 3 | P07451 | CA3 | AI62/PP34 | 1FLJ | ‒3.9758468 |
| T28 | Cyclin-dependent kinase 2 | P24941 | CDK2 | PP18 | 6ATH | ‒6.5244699 |
| T28 | Cyclin-dependent kinase 2 | P24941 | CDK2 | PP41 | 6ATH | ‒5.3482327 |
| T28 | Cyclin-dependent kinase 2 | P24941 | CDK2 | PP14 | 6ATH | ‒5.1597323 |
| T28 | Cyclin-dependent kinase 2 | P24941 | CDK2 | PP36 | 6ATH | ‒4.30864 |
| T30 | Cytochrome P450 1A1 | P04798 | CYP1A1 | PP32 | 6DWN | ‒5.7328925 |
| T30 | Cytochrome P450 1A1 | P04798 | CYP1A1 | AI60 | 6DWN | ‒5.5378819 |
| T31 | Cytochrome P450 1A2 | P05177 | CYP1A2 | AI28 | 2HI4 | ‒7.3450336 |
| T31 | Cytochrome P450 1A2 | P05177 | CYP1A2 | CC27 | 2HI4 | ‒6.9282684 |
| T31 | Cytochrome P450 1A2 | P05177 | CYP1A2 | AI36 | 2HI4 | ‒4.994256 |
| T31 | Cytochrome P450 1A2 | P05177 | CYP1A2 | PP20 | 2HI4 | ‒4.4958668 |
| T32 | Cytochrome P450 1B1 | Q16678 | CYP1B1 | CC38 | 6IQ5 | ‒10.099092 |
| T32 | Cytochrome P450 1B1 | Q16678 | CYP1B1 | CC33 | 6IQ5 | ‒8.5111952 |
| T32 | Cytochrome P450 1B1 | Q16678 | CYP1B1 | PP10 | 6IQ5 | ‒8.047123 |
| T32 | Cytochrome P450 1B1 | Q16678 | CYP1B1 | PP09 | 6IQ5 | ‒8.0444698 |
| T32 | Cytochrome P450 1B1 | Q16678 | CYP1B1 | PP11 | 6IQ5 | ‒7.8512955 |
| T32 | Cytochrome P450 1B1 | Q16678 | CYP1B1 | PP12 | 6IQ5 | ‒7.8441854 |
| T32 | Cytochrome P450 1B1 | Q16678 | CYP1B1 | AI28 | 6IQ5 | ‒7.5583858 |
| T32 | Cytochrome P450 1B1 | Q16678 | CYP1B1 | CC29 | 6IQ5 | ‒7.5107846 |
| T32 | Cytochrome P450 1B1 | Q16678 | CYP1B1 | AI27/PP02/CC28 | 6IQ5 | ‒7.4903784 |
| T32 | Cytochrome P450 1B1 | Q16678 | CYP1B1 | CC27 | 6IQ5 | ‒7.3440156 |
| T32 | Cytochrome P450 1B1 | Q16678 | CYP1B1 | AI60 | 6IQ5 | ‒6.8530407 |
| T34 | Cytochrome P450 2D6 | P10635 | CYP2D6 | PP14 | 6CSB | ‒5.7875323 |
| T35 | Cytochrome P450 3A4 | P08684 | CYP3A4 | CC27 | 6MA6 | ‒5.8861551 |
| T36 | Epidermal growth factor receptor | P00533 | EGFR | AI27/PP02/CC28 | 4V0G | ‒5.9841714 |
| T37 | Prothrombin | P00734 | F2 | AI107 | 6EPN | ‒8.7703018 |
| T37 | Prothrombin | P00734 | F2 | AI27/PP02/CC28 | 6EPN | ‒6.7194176 |
| T37 | Prothrombin | P00734 | F2 | PP18 | 6EPN | ‒6.1260571 |
| T42 | Lactoylglutathione lyase | Q04760 | GLO1 | CC38 | 4X2A | ‒6.825563 |
| T42 | Lactoylglutathione lyase | Q04760 | GLO1 | AI28 | 4X2A | ‒5.2546501 |
| T42 | Lactoylglutathione lyase | Q04760 | GLO1 | PP41 | 4X2A | ‒5.0670395 |
| T42 | Lactoylglutathione lyase | Q04760 | GLO1 | AI60 | 4X2A | ‒4.6537285 |
| T42 | Lactoylglutathione lyase | Q04760 | GLO1 | AI64 | 4X2A | ‒4.3709984 |
| T43 | G-protein coupled bile acid receptor 1 | Q8TDU6 | GPBAR1 | AI05 | 5OSB | ‒7.2971182 |
| T43 | G-protein coupled bile acid receptor 1 | Q8TDU6 | GPBAR1 | AI06 | 5OSB | ‒7.0067906 |
| T43 | G-protein coupled bile acid receptor 1 | Q8TDU6 | GPBAR1 | AI08 | 5OSB | ‒6.9686947 |
| T43 | G-protein coupled bile acid receptor 1 | Q8TDU6 | GPBAR1 | AI10 | 5OSB | ‒6.9686947 |
| T43 | G-protein coupled bile acid receptor 1 | Q8TDU6 | GPBAR1 | AI16 | 5OSB | ‒6.9686947 |
| T43 | G-protein coupled bile acid receptor 1 | Q8TDU6 | GPBAR1 | AI22_qt | 5OSB | ‒6.8831973 |
| T43 | G-protein coupled bile acid receptor 1 | Q8TDU6 | GPBAR1 | AI07 | 5OSB | ‒6.811522 |
| T53 | Interleukin-1 beta | P01584 | IL-1β | AI01 | 4DEP | ‒6.7696757 |
| T53 | Interleukin-1 beta | P01584 | IL-1β | AI03 | 4DEP | ‒5.8964744 |
| T54 | Interleukin-2 | P60568 | IL-2 | AI89 | 4YQX | ‒6.1749662 |
| T54 | Interleukin-2 | P60568 | IL-2 | AI93 | 4YQX | ‒6.1033737 |
| T54 | Interleukin-2 | P60568 | IL-2 | AI90 | 4YQX | ‒6.0446154 |
| T54 | Interleukin-2 | P60568 | IL-2 | CC38 | 4YQX | ‒5.4339032 |
| T54 | Interleukin-2 | P60568 | IL-2 | AI22_qt | 4YQX | ‒5.2682195 |
| T54 | Interleukin-2 | P60568 | IL-2 | AI47 | 4YQX | ‒5.2072554 |
| T54 | Interleukin-2 | P60568 | IL-2 | AI21_qt | 4YQX | ‒5.1139083 |
| T54 | Interleukin-2 | P60568 | IL-2 | AI42 | 4YQX | ‒4.97825 |
| T54 | Interleukin-2 | P60568 | IL-2 | AI45 | 4YQX | ‒4.9504733 |
| T54 | Interleukin-2 | P60568 | IL-2 | CC33 | 4YQX | ‒4.6616755 |
| T54 | Interleukin-2 | P60568 | IL-2 | PP36 | 4YQX | ‒4.0765986 |
| T55 | Leukotriene B4 receptor 1 | Q15722 | LTB4R | AI64 | 5X33 | ‒4.4258208 |
| T56 | Amine oxidase [flavin-containing] A | P21397 | MAOA | AI28 | 6EZZ | ‒6.1187067 |
| T57 | Amine oxidase [flavin-containing] B | P27338 | MAOB | AI60 | 6FVZ | ‒6.123919 |
| T57 | Amine oxidase [flavin-containing] B | P27338 | MAOB | AI61/PP40 | 6FVZ | ‒5.3824663 |
| T58 | Mitogen-activated protein kinase 14 | Q16539 | MAPK14 | PP14 | 1YQJ | ‒5.925612 |
| T58 | Mitogen-activated protein kinase 14 | Q16539 | MAPK14 | PP32 | 1YQJ | ‒5.464734 |
| T60 | Interstitial collagenase | P03956 | MMP1 | AI107 | 1FBL | ‒5.8512025 |
| T60 | Interstitial collagenase | P03956 | MMP1 | AI60 | 1FBL | ‒4.2860422 |
| T60 | Interstitial collagenase | P03956 | MMP1 | AI61/PP40 | 1FBL | ‒4.1799464 |
| T60 | Interstitial collagenase | P03956 | MMP1 | AI58 | 1FBL | ‒4.0021753 |
| T60 | Interstitial collagenase | P03956 | MMP1 | PP26 | 1FBL | ‒4.2788906 |
| T61 | 72 kDa type IV collagenase | P08253 | MMP2 | AI90 | 3AYU | ‒8.9635265 |
| T61 | 72 kDa type IV collagenase | P08253 | MMP2 | AI107 | 3AYU | ‒6.0359254 |
| T61 | 72 kDa type IV collagenase | P08253 | MMP2 | AI28 | 3AYU | ‒4.8741484 |
| T61 | 72 kDa type IV collagenase | P08253 | MMP2 | AI27/PP02/CC28 | 3AYU | ‒4.8642421 |
| T61 | 72 kDa type IV collagenase | P08253 | MMP2 | PP26 | 3AYU | ‒4.5178051 |
| T61 | 72 kDa type IV collagenase | P08253 | MMP2 | PP25 | 3AYU | ‒4.4763627 |
| T61 | 72 kDa type IV collagenase | P08253 | MMP2 | AI58 | 3AYU | ‒3.927405 |
| T61 | 72 kDa type IV collagenase | P08253 | MMP2 | AI56 | 3AYU | ‒3.6693187 |
| T62 | Stromelysin-1 | P08254 | MMP3 | AI107 | 3OHL | ‒7.6850858 |
| T62 | Stromelysin-1 | P08254 | MMP3 | AI27/PP02/CC28 | 3OHL | ‒6.8599362 |
| T62 | Stromelysin-1 | P08254 | MMP3 | AI73/PP43 | 3OHL | ‒5.1445894 |
| T64 | Nuclear factor NF-κB p105 subunit | P19838 | NF-κB1 | CC43 | 5T8Q | ‒6.4699578 |
| T65 | NAD(P)H dehydrogenase [quinone] 1 | P15559 | NQO1 | CC43 | 5FUQ | ‒6.4538054 |
| T65 | NAD(P)H dehydrogenase [quinone] 1 | P15559 | NQO1 | PP41 | 5FUQ | ‒5.6699967 |
| T65 | NAD(P)H dehydrogenase [quinone] 1 | P15559 | NQO1 | PP36 | 5FUQ | ‒5.2224779 |
| T65 | NAD(P)H dehydrogenase [quinone] 1 | P15559 | NQO1 | AI37 | 5FUQ | ‒4.8933864 |
| T65 | NAD(P)H dehydrogenase [quinone] 1 | P15559 | NQO1 | AI36 | 5FUQ | ‒4.7754536 |
| T68 | Peroxisome proliferator-activated receptor gamma | P37231 | PPARγ | AI107 | 6C5Q | ‒8.1971264 |
| T71 | Transcription factor p65 | Q04206 | RELA | CC33 | 1K3Z | ‒7.0631828 |
| T71 | Transcription factor p65 | Q04206 | RELA | AI60 | 1K3Z | ‒5.4502521 |
| T77 | Sodium/bile acid cotransporter | Q14973 | SLC10A1 | AI07 | 6QD5 | ‒8.49016 |
| T77 | Sodium/bile acid cotransporter | Q14973 | SLC10A1 | AI06 | 6QD5 | ‒8.3634224 |
| T77 | Sodium/bile acid cotransporter | Q14973 | SLC10A1 | AI05 | 6QD5 | ‒8.0610256 |
| T77 | Sodium/bile acid cotransporter | Q14973 | SLC10A1 | AI22_qt | 6QD5 | ‒7.9677043 |
| T77 | Sodium/bile acid cotransporter | Q14973 | SLC10A1 | AI10 | 6QD5 | ‒7.9261217 |
| T77 | Sodium/bile acid cotransporter | Q14973 | SLC10A1 | AI08 | 6QD5 | ‒7.8954577 |
| T78 | Ileal sodium/bile acid cotransporter | Q12908 | SLC10A2 | AI08 | 3UBD | ‒8.2698698 |
| T78 | Ileal sodium/bile acid cotransporter | Q12908 | SLC10A2 | AI06 | 3UBD | ‒7.7178326 |
| T78 | Ileal sodium/bile acid cotransporter | Q12908 | SLC10A2 | AI05 | 3UBD | ‒7.558876 |
| T82 | Tumor necrosis factor receptor superfamily member 1A | P19438 | TNFRSF1A | AI65 | 1EXT | ‒5.4425287 |
| T82 | Tumor necrosis factor receptor superfamily member 1A | P19438 | TNFRSF1A | AI60 | 1EXT | ‒5.0871925 |
| T82 | Tumor necrosis factor receptor superfamily member 1A | P19438 | TNFRSF1A | AI64 | 1EXT | ‒4.8801584 |
| T86 | Vitamin D3 receptor | P11473 | VDR | AI08 | 3WWR | ‒11.500716 |
| T86 | Vitamin D3 receptor | P11473 | VDR | AI05 | 3WWR | ‒10.72134 |
| T86 | Vitamin D3 receptor | P11473 | VDR | AI06 | 3WWR | ‒10.535404 |
| T86 | Vitamin D3 receptor | P11473 | VDR | PP09 | 3WWR | ‒7.933929 |
| T87 | Vascular endothelial growth factor A | P15692 | VEGFA | AI89 | 4GLS | ‒8.5744066 |
| T87 | Vascular endothelial growth factor A | P15692 | VEGFA | AI93 | 4GLS | ‒8.3576736 |
| T87 | Vascular endothelial growth factor A | P15692 | VEGFA | AI90 | 4GLS | ‒8.126845 |
| T87 | Vascular endothelial growth factor A | P15692 | VEGFA | CC29 | 4GLS | ‒5.6705046 |
| T87 | Vascular endothelial growth factor A | P15692 | VEGFA | PP11 | 4GLS | ‒5.5328727 |
| T87 | Vascular endothelial growth factor A | P15692 | VEGFA | PP12 | 4GLS | ‒5.5310359 |
| T87 | Vascular endothelial growth factor A | P15692 | VEGFA | AI42 | 4GLS | ‒5.4957933 |
| T87 | Vascular endothelial growth factor A | P15692 | VEGFA | PP10 | 4GLS | ‒5.3272915 |
| T87 | Vascular endothelial growth factor A | P15692 | VEGFA | CC27 | 4GLS | ‒5.2873363 |
| T87 | Vascular endothelial growth factor A | P15692 | VEGFA | PP25 | 4GLS | ‒5.0034289 |

**Table S5** Detailed information of four significant metabolism pathways

| Pathway | Total | Hits | Raw p | LOG(p) | Holm adjust | FDR | Impact |
| --- | --- | --- | --- | --- | --- | --- | --- |
| Glycerophospholipid metabolism | 30 | 5.00 | ≤ 0.01 | 10.71 | 0.00 | 0.00 | 0.11 |
| Glutathione metabolism | 26 | 2.00 | 0.04 | 3.17 | 1.00 | 0.85 | 0.42 |
| Cysteine and methionine metabolism | 28 | 2.00 | 0.05 | 3.03 | 1.00 | 0.85 | 0.12 |
| Linoleic acid metabolism | 5 | 1.00 | 0.04 | 2.77 | 1.00 | 0.85 | 1.00 |

Note: “Total” is the total number of differential metabolites species in the pathway; “hits” is the actually matched number from the 18 differential biomarkers; “raw *P*” is the original *P* calculated from the enrichment analysis; “impact” is the pathway impact value calculated from pathway topology analysis.
