# Supplementary figures and images for: Deciphering the Underlying Mechanisms of Formula Le-Cao-Shi Against Liver Injuries by Integrating Network Pharmacology, Metabonomics, and Experimental Validation
Source: Front Pharmacol. 2022 Apr 25;13:884480. doi: 10.3389/fphar.2022.884480 (PMC9081656; doi:10.3389/fphar.2022.884480)

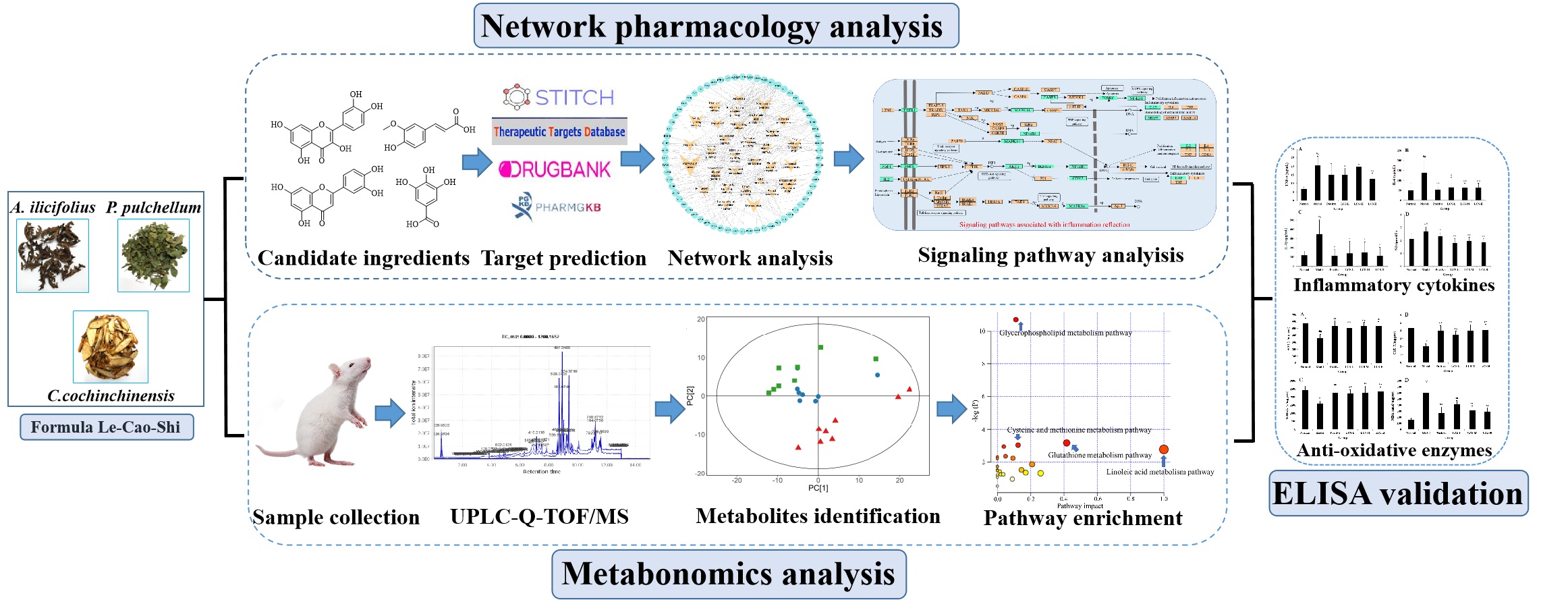

Supplement: Supplementary file 2 [file Image1.JPEG]
